# Access to Vaccination among Disadvantaged, Isolated and Difficult-to-Reach Communities in the WHO European Region: A Systematic Review

**DOI:** 10.3390/vaccines10071038

**Published:** 2022-06-28

**Authors:** Winifred Ekezie, Samy Awwad, Arja Krauchenberg, Nora Karara, Łukasz Dembiński, Zachi Grossman, Stefano del Torso, Hans Juergen Dornbusch, Ana Neves, Sian Copley, Artur Mazur, Adamos Hadjipanayis, Yevgenii Grechukha, Hanna Nohynek, Kaja Damnjanović, Milica Lazić, Vana Papaevangelou, Fedir Lapii, Chen Stein-Zamir, Barbara Rath, for the ImmuHubs Consortium

**Affiliations:** 1 Vienna Vaccine Safety Initiative e.V., 10437 Berlin, Germany; wce2@leicester.ac.uk (W.E.); samy.awwad7@gmail.com (S.A.); 2 Department of Epidemiology and Public Health, University of Nottingham, Nottingham NG5 1PB, UK; 3 College of Life Sciences, University of Leicester, Leicester LE5 4PW, UK; 4 ImmuHubs Consortium, Coordinating Entity: Vienna Vaccine Safety Initiative e.V., 10437 Berlin, Germany; arja.krauchenberg@gmail.com; 5 Stanford University, Palo Alto, CA 94305, USA; 6 European Parents Association, 1000 Brussels, Belgium; 7 Young European Academy of Paediatrics, 1000 Brussels, Belgium; norakarara@gmail.com; 8 Evangelical Hospital Queen Elisabeth Herzberge, 10365 Berlin, Germany; 9 European Academy of Paediatrics, 1000 Brussels, Belgium; lukaszdembinski@gmail.com (Ł.D.); zgrosman@netvision.net.il (Z.G.); deltorso@gmail.com (S.d.T.); hansjdornbusch@gmail.com (H.J.D.); amneves37@gmail.com (A.N.); sian.copley@gmail.com (S.C.); drmazur@poczta.onet.pl (A.M.); adamos@paidiatros.com (A.H.); ineugenius@gmail.com (Y.G.); vpapaev@gmail.com (V.P.); dr.fedirlapiy@gmail.com (F.L.); 10 Department of Pediatric Gastroenterology and Nutrition, Medical University of Warsaw, 02-091 Warsaw, Poland; 11 Adelson School of Medicine, Ariel University, Ariel 40700, Israel; 12 Finnish Institute for Health and Welfare, FI-00271 Helsinki, Finland; hanna.nohynek@thl.fi; 13 Faculty of Philosophy, University of Belgrade, 11000 Beograd, Serbia; kdamnjan@f.bg.ac.rs; 14 Faculty of Philosophy, University of Novi Sad, 21000 Novi Sad, Serbia; milica.lazic@ff.uns.ac.rs; 15 Jerusalem District Health Office, Jerusalem 94341, Israel; chen.zamir@mail.huji.ac.il

**Keywords:** Europe, disadvantaged groups, health services, immunisation programs, inequalities, migration, vaccination

## Abstract

Vaccination has a significant impact on morbidity and mortality. High vaccination coverage rates are required to achieve herd protection against vaccine-preventable diseases. However, limited vaccine access and hesitancy among specific communities represent significant obstacles to this goal. This review provides an overview of critical factors associated with vaccination among disadvantaged groups in World Health Organisation European countries. Initial searches yielded 18,109 publications from four databases, and 104 studies from 19 out of 53 countries reporting 22 vaccine-preventable diseases were included. Nine groups representing the populations of interest were identified, and most of the studies focused on asylum seekers, refugees, migrants and deprived communities. Recall of previous vaccinations received was poor, and serology was conducted in some cases to confirm protection for those who received prior vaccinations. Vaccination coverage was lower among study populations compared to the general population or national average. Factors that influenced uptake, which presented differently at different population levels, included health service accessibility, language and vaccine literacy, including risk perception, disease severity and vaccination benefits. Strategies that could be implemented in vaccination policy and programs were also identified. Overall, interventions specific to target communities are vital to improving uptake. More innovative strategies need to be deployed to improve vaccination coverage among disadvantaged groups.

## 1. Introduction

In recent years, Europe has been dealing with increasing rates of vaccine-preventable diseases (VPD) and complex determinants of vaccination, which have contributed to the stagnation of childhood and adult vaccine uptake and increased disease incidences that pre-existed and go beyond the COVID-19 pandemic [1,2]. Population dynamics significantly influence healthcare access, and several communities have been identified to be of particular concern. These groups include populations that are disadvantaged and difficult to reach due to socioeconomic (disenfranchised populations), cultural/religious (isolated and closed communities) and geographic reasons (selected ethnicities, border populations and economic travellers) [3]. These populations may differ in some of their characteristics, such as migration status, personal beliefs, geographical location or deprivation, but they all have difficulties accessing and being reached by health services and show poorer health outcomes compared to the average national population [4].

Although vaccination uptake for certain diseases is already sub-optimal in general populations, this is even lower among disenfranchised and disadvantaged groups. Reasons for vaccine non-acceptance or incompletion (i.e., not receiving all doses of a multi-dose vaccine) vary among these groups. Some examples include poor immunisation rates in refugees’ countries of origin, cultural, religious and personal beliefs and healthcare system barriers [3,5,6]. To tackle vaccination in Europe, the European Vaccine Action Plan 2015–2020 (EVAP) was developed to address the specific needs and challenges related to immunisation in the WHO European Region [7]. Still, vaccination policies, which inform how governments prevent the spread of infectious disease through vaccination, vary across the World Health Organization (WHO) European Region member states. Common barriers to implementing and utilising immunisation services have also been identified across member countries [8,9,10]. However, the diversity among different disenfranchised and disadvantaged groups poses further challenges in implementing effective vaccine programs and immunisation strategies, as this often requires special design efforts, vaccine policies, decision processes and outcomes, which vary widely across European countries [9,10].

Accessibility to health services, including vaccination, is a complex and multifaceted issue. Among disenfranchised, disadvantaged and difficult-to-reach groups, healthcare access is particularly affected by a lack of familiarity with the healthcare system, language barriers and an absence of culturally appropriate care. It has also been recognised that some populations, particularly new and temporary migrants, are often unaware of their legal status and hence might miss out on healthcare opportunities, including access to vaccination services [11]. While many studies have documented vaccination coverage rates (VCRs) among disenfranchised and disadvantaged populations as separate groups [5], only a few have been conducted to explore the reasons behind the disparities across these groups collectively. Previous research in Europe has indicated that these groups are more likely to accept vaccination with the implementation of effective engagement aimed at increasing vaccine uptake [3,5,12]. Understanding how to encourage uptake is an important public health aim; therefore, by understanding the barriers, policymakers and healthcare providers may be better able to address concerns and develop strategies to increase vaccination rates effectively [13]. Hence, we conducted this review study to provide an overview of vaccination coverage, accessibility, underlying factors and critical issues related to disadvantaged, isolated and difficult-to-reach communities in the WHO Europe Region. The study findings highlight factors that need to be considered when developing vaccination programs for these communities.

## 2. Materials and Methods

The primary objective of this review was to identify and synthesise quantitative and qualitative studies that examined vaccination coverage, uptake, barriers, facilitators, accessibility and challenges among disadvantaged, isolated and difficult-to-reach communities in WHO European Region countries.

The study protocol was registered on PROSPERO (CRD42020192530) [14], and the review followed the Preferred Reporting Items for Systematic Reviews and Meta-Analyses (PRISMA) guidelines [15]. Before performing the study, similar reviews were searched, but none were found. In consultation with a medical librarian, we conducted a comprehensive search of peer-reviewed literature. A review of published literature was performed in order to identify related publications describing studies related to the research question between 2015 and March 2022. The initial search was run in November 2020 and was updated on 5 March 2022. The update allowed the inclusion of relevant COVID-19 vaccine coverage studies.

### 2.1. Eligibility Criteria

Only studies presenting information on populations in 53 WHO European Region countries from 2015 were considered for the review; this included studies conducted in previous years but also those which contained data from the year 2015 to reflect the commencement of the European vaccine policy implementation [7]. The specific population of interest was disadvantaged and difficult-to-reach groups of all ages and genders who are at risk of VPDs and who would require vaccination. The definition of disadvantaged groups for this study was based on socioeconomic factors and location, not on clinical factors. Disadvantaged groups considered were those with a higher risk of social exclusion, discrimination and violence than the general population. Hence, the target study group for this review included disenfranchised populations, isolated or closed communities, ethnic minorities, cross-border populations and migrants. Disenfranchised populations are defined as people deprived of some rights and privileges of full participation in society. Isolated or closed communities are groups who intentionally limit links with outsiders and outside communities for religious, ethnic or geographic reasons. Ethnic minorities are groups with different national or cultural traditions from the main population in any country. While cross-border populations are people who move to different countries for work purposes, this group also includes migrants who move from one country and settle in another country for any reason. Some population groups have more than one of the listed disadvantage characteristics; therefore, this review will present the relevant groups as they are reported and presented within the included studies. The study exclusion criteria included groups exempt from specific vaccines or those generally recommended to have specific vaccines due to their age and higher susceptibility due to certain conditions, including known risk groups (i.e., children, elderly, pregnant women, immunocompromised patients, e.g., HIV), people specifically targeted for certain vaccinations (e.g., men who have sex with men), those outside the WHO European Region and leisure travellers.

The vaccines of interest for the review were those most commonly recommended to the general population, including diphtheria, pertussis, tetanus, poliovirus, measles, tuberculosis and influenza. Data from individual, group, population and facility levels were assessed. The study explored vaccine services and immunisation accessibility and measured the beliefs and experiences of disadvantaged groups and vaccination providers as reported in the papers analysed. Outcomes evaluated were the proportion of the study population who received vaccinations, those who had access to vaccination services, the available access routes for vaccinations, vaccine coverage rates (VCRs), VPD outbreaks, factors that influenced vaccination consideration (barriers and facilitators) and decisions associated with vaccination provision consideration (drivers of vaccination services).

The outcomes of interest were the proportion of specific disadvantaged groups accessing vaccination services, available vaccination access routes, vaccine coverage rates and the factors that influenced vaccination consideration. Only quantitative data (or inferred quantitative information in qualitative studies) reporting coverage from the year 2015 onwards were included.

### 2.2. Search Strategy

A search was conducted using Embase, MEDLINE, Scopus and Web of Science databases, and the search strategy was designed by a medical librarian using a combination of MeSH terms and keywords. The search subject headings were based on three key concepts: vaccination, disadvantaged populations and the WHO European countries (see Supplementary File S1). Only publications available in the English language were considered. All records were retrieved and imported into the Rayyan review manager for screening based on the eligibility criteria [16]. Duplicates were removed, and an assessment of titles and abstracts was conducted for the remaining publications. A full-text review was then conducted, and the reference lists of final selected studies and related systematic reviews were examined for relevant articles.

### 2.3. Study Selection

We obtained the titles and abstracts of all studies resulting from the search conducted. The literature search was carried out by WE; article screening, data extraction and quality assessment were performed by WE, SA and NK. Abstracts were included for full-text review if they appeared to meet all inclusion criteria and none of the exclusion criteria. Study titles and abstracts needed only to be deemed potentially relevant by one reviewer in order to move on to the full-text screening stage. Studies were included if they reported quantitative individual-level vaccination status, including coverage, uptake and antibody protection. No disagreements arose at the final inclusion stage that necessitated an additional reviewer.

### 2.4. Data Extraction and Analysis

Data from all included studies were extracted into a spreadsheet in Excel. Key variables extracted included: author(s), year, country, intervention details, study population, design, setting and outcomes. Two reviewers conducted data screening and extraction, and disagreements were resolved through discussions with a third reviewer. Only descriptive data analysis was performed as the studies were highly heterogeneous, and meta-analysis was not possible.

### 2.5. Study Appraisal

Quality assessment was conducted using the JBI checklist [17] by two reviewers (WE and SA). JBI quality assessment is not judged as a numerical scoring of the checklist components; therefore, this review had a subjective element to the grading decision. Studies were graded based on how many of the assessment requirements were met: <60% requirements (low), 60–79% (medium) and ≤80% (high). The high cut-off for the low range was used to factor in limitations of studies which are often not reflected in quality assessments, e.g., variation in populations and study time points.

## 3. Results

### 3.1. Study Selection

**Figure 1:**
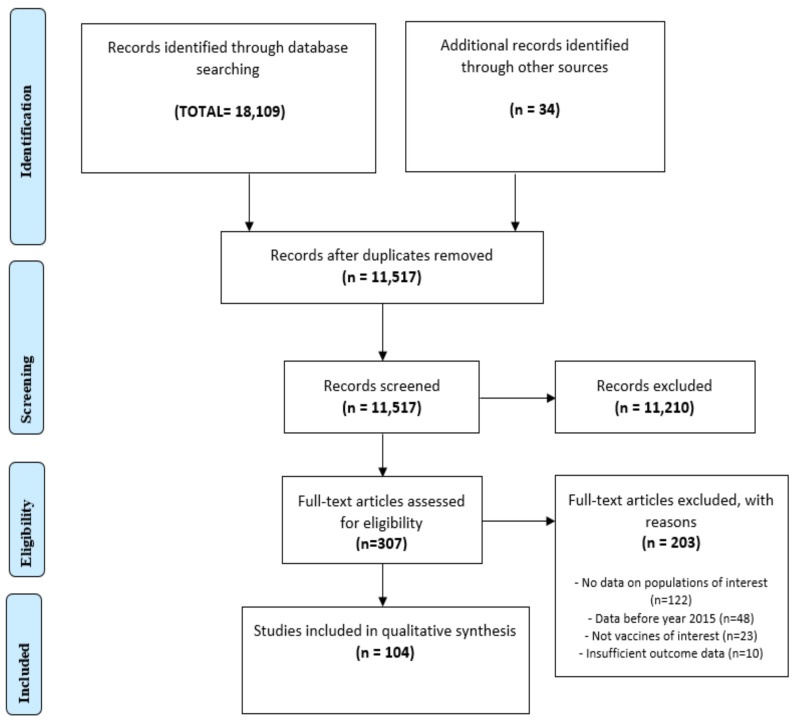
Flow diagram of study inclusion.

A total of 18,109 search results were identified from four databases and 34 from reference lists, grey literature and preprints (Figure 1). After removing duplicates, titles and abstracts, 11,519 studies were screened, and 307 studies were identified as eligible for full-text review. A total of 203 studies that had no information on the populations of interest, did not report relevant vaccines or included only information before 2015 were excluded. Finally, 104 publications were included in this review, and this included 19 studies on COVID-19 vaccine uptake. Most of the studies were cross-sectional, and the general quality range was low to medium (low-quality, 44 studies; medium-quality, 36 studies; and high-quality, 24 studies). The main differences between the study quality levels were related to vaccination exposure criteria (e.g., self-reported, laboratory testing) and depth of statistical analysis, e.g., most high-quality studies conducted an appropriate statistical analysis that included accounting for confounding factors such as age, gender and country of origin. The quality assessment showed an overview of the methodological process in the included studies and was not used to interpret the finding so as not to limit the interpretation of the information provided.

### 3.2. Characteristics of Included Studies

A detailed description of the characteristics and quality of the included studies conducted in 19 of the 53 WHO European countries identified is provided in Supplementary Files S2 and S3. The countries were Belgium, Denmark, Finland, France, Germany, Greece, Israel, Italy, The Netherlands, Norway, Poland, Romania, Slovakia, Spain, Sweden, Switzerland, Turkey, Ukraine and the United Kingdom (England, Scotland and Wales). Different vaccines were reported for 22 infectious diseases. The vaccines included those protecting against a single disease, for example, oral polio vaccine (OPV), pneumococcal vaccine (PCV) and COVID-19, as well as combination vaccines and multiple doses, such as the DTaP-IPV-Hib vaccine used against diphtheria, tetanus, pertussis, polio and haemophilus influenza type b (Hib) and DTaP4 (“4” here means four doses of the single vaccine are needed for vaccine course completion). A summary of specific vaccines and countries represented in the included studies is shown in Table 1. Studies not reporting specific vaccines are not included in the table (refer to Supplementary File S2 for more details).

Nine groups representing the populations of interest were reported: adopted children, asylum seekers, expatriates, internally displaced persons, religious groups, migrants, refugees, socioeconomically deprived populations, cross-border workers and Roma communities. Most studies were cross-sectional, and data were collected using qualitative and quantitative methods. Data in 36 studies included information before 2015 but ended between 2015 and 2019, and the longest study period was 19 years (1999–2018).

#### 3.2.1. Coverage for Non-COVID-19 Vaccines

The non-COVID-19 studies included (n = 87 studies) investigated vaccine coverage and uptake for key recommended vaccines. There were wide VCR ranges across the different countries and population groups. Non-COVID-19 vaccines included influenza, measles, diphtheria, pertussis, tetanus, polio, mumps, rubella, pneumococcal disease, Hib and hepatitis B (HBV) (see Supplementary File S2). Most studies focused on routine childhood vaccines (n = 38) compared to vaccines for all ages and life course immunisation (36 studies) and adult vaccines only (24 studies). Insufficient vaccination coverage was reported for certain diseases, especially for second and subsequent doses, leading to incomplete and non-up-to-date vaccination status [48,64,65,89,96,100,106,109]. Among migrant populations, this implied a need for mass vaccination and booster campaigns.

A number of studies examined seroprevalence as a correlate of vaccine protection, and varying antibody levels were detected for those who responded as having received specific vaccinations [44,45,52,57,60,61,63,78,83,88,90,91,97,99]. For example, one study observed that, among 200 migrant children, for protection against HBV, only 118 (59%) had anti-HBs ≥1000 UI/L compared to 23 (11%) having no detectable antibodies (<10 IU/L) [57]. Some serological studies also noted false-negative results; for instance, some non-tuberculosis patients had false-positive tuberculin skin tests despite being BCG-vaccinated [19]. Regardless of some variance in accuracy and specificity, serology served as an approximation of protection levels across different migrant groups [44,45,90].

The vaccine with the highest coverage of 90% and above was MMR, which often included incomplete MMR dose uptake [48,53,54,55,82,90]. However, uptake reduced with subsequent does; for instance, among the Irish travelling community in the UK, coverage of MMR1 and MMR2 was 54.0% and 46.7%, respectively. This reduction was also reflected in the comparison non-traveller group; however, their uptake was higher (MMR1: 95.5%, MMR2: 89.3%) [43].

Various other vaccines against respiratory infections were reported in the studies. The BCG vaccines reported were only offered to children, and the VCR ranged from as low as 57.6% among asylum seekers in Germany to 84.6% in settled Polish migrant children in Scotland [21,22]. However, when assessing self-report immunisation status for tuberculosis, only 18.8% of homeless migrants reported being vaccinated [32]. Influenzae VCR was generally higher among older populations; however, living in low-SES regions decreased the odds of being vaccinated among older adults (OR = 0.75 vs. 0.93 for higher SES) [76]. Vaccine against Haemophilus influenzae type b (HiB) was often given as a combination (DTaP-IPV-Hib). However, specific coverage was identified as being low, especially among refugees in camp settings, as shown in Greece, where high rates of no Hib dose were identified in some camps, with camps with fewer children having lower coverage (no dose = 69.7% for camp size 1–99 children, 56.2% for camp size ≥ 100 children) [48]. Pneumonia vaccines were less commonly reported, and wide gaps identified populations, e.g., uptake in the Irish traveller community compared to non-travellers in the UK was 47.4% vs. 89.2% [43]. Additionally, the meningococcal vaccine was reported only in a few countries, and uptake varied widely; for instance, a VCR of 0% was reported among teenage Irish travellers compared to the national average of 58.8%. However, high coverage was reported when the onsite MCV vaccine was provided to asylum seekers in Italy (86% of children vaccinated) [43,50]. The only study reporting on whooping cough did not provide disaggregated data for the specific vaccine.

Diphtheria, tetanus and pertussis vaccination were often offered in combination. Diphtheria specific uptake report was as low as 23.9% among refugees in Germany and high as 82% among asylum seekers in The Netherlands [44,45]. For tetanus, low coverage was noted among adults compared to children, e.g., among asylum seekers in Germany the VCR among children was 40.7% compared to adults 28% [58]. Although pertussis VCR was mostly low, one study in Italy showed comparably high uptake, and timeliness in different socioeconomic regions (uptake: most deprived 97.1%, least deprived 98.2%) (received three doses on time in 2017: most deprived 76.9%, least deprived 87.3%) [96].

Hepatitis vaccines for types A, B and C were given to all age groups, but the coverage and protection were often low, e.g., self-reported Hep-A immunisation among migrants (homeless) in Italy was 15.6%, Hep-B protection was 8% among Iraq asylum seekers in The Netherlands; and Hep-C, the least commonly reported, had a protection level of 1.8% [32,44,60]. The polio vaccine was also often provided as a combination (DTaP-IPV-Hib), and some studies specifically reported on vaccines for the different types, and coverage was often high, as shown among asylum seekers in The Netherlands (polio type 1, 91%; type 2, 95%; type 3, 82%) [44]. The rotavirus vaccine was reported for only children, and low coverage was found in traveller communities (47.6%) and reduced uptake with subsequent dosage in disadvantaged areas (most deprived: first dose, 90.6%; second dose, 84.9%) [100]. Protection against shingles and varicella infections among migrants was high, for instance, seroprotection in Syrian migrants in Turkey (91.4%) and asylum seekers in The Netherlands (96%), but coverage by deprivation regions in the UK was lower (most deprived, 54.1%; least deprived, 64.1%) [23,44,101].

Overall, as observed for all vaccines identified in this review, coverage among the populations studied was below standard herd immunity thresholds with wide variation between locations, as shown in a study that reported the prevalence of protective antibodies (seroprotection) against selected thresholds and different countries [44].

#### 3.2.2. COVID-19 Vaccine Coverage

The 19 included studies on COVID-19 vaccines from four countries (Israel, Italy, Norway and the UK) are presented in Supplementary File S3. Two studies also reported on other vaccines (influenza, Hep-A, Hep-B and TB), which are shown only in Supplementary File S2 [25,32]. Studies in Italy showed moderate willingness to be vaccinated amongst homeless migrants (64.3%), but also low VCRs among asylum seekers (28.9%), irregulars (15.7%) and holders of other types of residency permits (38.5%) [25,42]. Ethnicity and deprivation were the two most common factors reported to influence COVID-19 vaccine uptake. VCRs amongst people previously infected with COVID-19 were not reported in the included studies, although one study observed higher vaccine hesitancy that was presumed to be associated with a substantially higher rate of prior COVID-19 infection [37]. Similar to other vaccines, the influence of religion on vaccine uptake was intertwined with both factors. For instance, in Israel, vaccine uptake was lower in towns with greater Arab and ultra-Orthodox Jewish populations compared to the general Jewish population [35]. Evidence was shown in the study reporting coverage of Arabs (64.4%), compared to ultra-Orthodox Jewish (46.7%), mixed religions (62.7%) and general Jewish (80.1%) [30], and uptake increased with improved residential socioeconomic status (SES). Additionally, although COVID-19 vaccine boosters (given after two doses of the basic series) are still underway, one study showed a gradual decline in receipt of subsequent doses across all groups (ethnic and SES). The study reported a correlation between COVID-19 vaccine uptake and SES category for different doses (Dose 1 (R2 = 0.4331), Dose 2 (R2 = 0.542), Dose 3 (R2 = 0.8416)] [39]. The study in Norway showed a lower vaccine uptake among children from foreign-born parents (73%) compared to Norwegian-born parents (93%) [33]. Similarly, all studies in the UK showed a significant influence of minority ethnicity on vaccine uptake among the general population and healthcare workers [31,34]. One UK study also reported that South Asian, black African and other ethnicities born in the UK had lower vaccination rates than their counterparts born abroad [28]. Although all minority ethnic groups had lower uptake compared to white British ethnicity (the majority population), the lowest were noted to be among black Africans (OR = 5.36; 95% CI: 5.32–5.40) and black Caribbeans (OR = 6.93; 95% CI: 6.87–6.98), as of June 2021. In addition, the level of deprivation significantly influenced COVID-19 vaccines, even among healthcare workers, as shown in the study reporting the odds of being vaccinated versus unvaccinated in the most deprived areas compared to the least deprived areas (OR = 0.50 (CI: 0.44–0.57), *p* < 0.0001), indicating a 50% lower likelihood of vaccine uptake in most deprived areas [31].

#### 3.2.3. Vaccine Coverage Population-Level Insight

Fewer studies were identified for people who migrated voluntarily compared to forced migrant groups. From the non-COVID-19 vaccines, this included economic migrants and cross-border populations (n = 18) compared to forced migrant groups, i.e., refugees/asylum seekers (n = 34), while COVID-19 vaccines studies were predominantly on disadvantaged areas and ethnic minority groups. Distinctions regarding the duration of stay, which is especially important for refugees/asylum seekers in the host countries, were rarely reported. Several migrants reported being vaccinated in their countries of origin [47,107]. However, the majority, particularly forced migrants, could not recall previous vaccinations received [44,47,57] and needed to be (re)vaccinated in their host country [47]. Hence, laboratory tests confirming immunisation or protection were often considered. Furthermore, in some locations with refugees, such as Greece, coverage was still low even after mass vaccination. For example, a mass child vaccination campaign against hepatitis A at five camps in Greece reached only 64.4% of the target population [62].

At a national level, most migrant communities did not have an immunisation status comparable to that of permanent residents, as shown in Italy, where rubella vaccination among immigrant women was nearly half that of Italian women (25.2% vs. 40.4%) [84] and measles vaccine coverage differences observed in Norway between children born to Somali parents (85%) and the national average (96%) [110]. Additionally, differences in vaccine uptake by countries of origin and specific demographic groups were also highlighted. For instance, two studies reporting seroprotection against tetanus and polio for migrants by WHO region showed that protective antibodies were high in migrants from the African region—AFR (tetanus, 28.2%; three polio types, 79.2%) and Eastern Mediterranean Region—EMR (tetanus, 28%; three polio types, 81.2%). In contrast to the high seroprevalences, vaccination uptake was often low in current countries of residence in Europe. For instance, amongst asylum-seeking children in Denmark, the least likely to be vaccinated were those from Afghanistan (57%) and Eritrea (54%) compared to those from Syria (28%), Russia (39%) or Somalia (24%) and Palestine (18%) [49]. In Israel, reported influenza vaccination rates were highest among children 1–4 years of age in both Jewish and Arab populations [72], but, generally, vaccination rates in these communities were lower than in Jerusalem [53].

Among the different community groups assessed, VCRs in religious groups showed de-creased vaccination coverage and vaccination delays in Jewish Ultra-Orthodox communities in the Jerusalem district in Israel. After vaccination campaigns, the coverage in these communi-ties increased to 82% DTaP4 and 94% MMR1/MMRV1 vaccine coverage in children, close to the mean district’s coverage (DTaP4, 89%; MMR1/ MMRV1, 96%); the aggregated VCR for children up to 7 years old showed overall adequate uptake with significantly lower coverage rates in Jewish Ultra-Orthodox groups [53]. Two other studies showed lower vaccination rates (<40%) among similar groups in Israel, including Arabs [72,111]. Another study in the United Kingdom reported a slightly similar pattern in crude vaccine uptake across different years among Jewish and Muslim people [112]. Among minority and religious groups in general, including ethnic minorities, Roma, Jewish and Arab communities had lower vaccination coverage rates compared to the general (local) population [42,54,69,70,72,76,86,90,101,109]. Vaccination coverage by disadvantaged areas revealed significant variation and association between level of deprivation and uptake. For instance, in the UK, crude influenzae vaccine uptake in the year 2015/16 in the most deprived areas compared to the least deprived areas was 26.7% vs. 39.5%, and the gap between the groups widened in the following season (year 2016/17, 29.0% vs. 42.9%) [112]. The same study also reported the impact of deprivation in combination with ethnicity and religion on vaccine uptake [112].

At an individual level, among undocumented children in The Netherlands, coverage was higher among those attending schools (88%) compared to non-school-attending children (50%) [113]. Additionally, gender differences were also observed; for example, one study reported higher rates of unvaccinated boys or boys with unknown immunisation status (37%) compared to girls (27%) [49]. Another study also showed younger parents and parents with fewer children were more likely to vaccinate their children compared with those who had more children (mean = 2.3 and 2.7, respectively; *p* < 0.05) [77].

### 3.3. Factors That Influence Considerations around Vaccination

Various factors influenced the availability of vaccines and uptake considerations among disadvantaged groups. These included issues related to access, affordability, awareness, acceptance and activation, as reported in the study by Bell et al. (2020) [18]. For instance, “access” was related to health service accessibility and acceptability and language and literacy, while “acceptance” was influenced by perceptions around disease severity, vaccination benefits and trust in vaccination and health services [18]. Awareness of disease severity, related vaccines and vaccine schedules influenced vaccine acceptance and uptake. Similar factors were grouped into three areas, sociopsychological, health-services-related and vaccine perceptions, by Stein-Zamir et al. (2017) and further stratified by population levels, presenting a different perspective, as described by Letley et al. (2018). Population-level considerations included the society-level (opportunity), community-level (support) and individual-level (personal motivation) factors [109]. The drivers, barriers and facilitators for vaccination coverage and uptake from the included studies are summarised under four levels: national, health services, community and individual levels (Table 2).

#### 3.3.1. Access to Immunisation Services

Providers of vaccines to people on the move are often the national public health authorities, healthcare workers or NGOs reaching out to refugees upon arrival in the host country [47]. Among asylum seekers, adult vaccination was mainly performed by NGOs and national healthcare employees, while children were vaccinated primarily by NGOs, local public healthcare facilities or doctors at asylum centres [47,62]. Newly arrived migrants identified in this review preferred receiving vaccination through public healthcare systems and NGOs. Constant monitoring of vaccine coverage in some settings, for instance, in camps, using a specially designed vaccination registry, was not always sustainable [48]. However, studies showed that incomplete vaccinations decreased when individuals had access to a summary of their risk factors in a single “Green Book” [65]. This observation supported other findings that show regular reminders and the help of local medical facilities in the destination area could enhance awareness and compliance with infectious disease prevention measures [106]. An identified approach that considered individual factors was shown in the study that adapted a vaccination “monthly appointment” strategy directly managed by the staff of local public health companies (ASL Rome-F) to a single “on arrival appointment” managed by the physicians of the internal healthcare facility (IHF), under the supervision of ASL Rome-F [55]. When physicians were able to monitor individual patients during a given observation period, it resulted in an increase in vaccination rates. Similar approaches that led to a VCR increase included the coordination of various partners, the provision of vaccines and other materials by the health department and cooperation with the district medical profession [81]. For COVID-19 vaccinations, access was high, but uptake in hospitals was considered a limitation, especially among elderly populations; hence, wide-range vaccination campaigns and delivery through mass vaccination centres were initiated to boost rapid uptake [24,38,40,42].

#### 3.3.2. Vaccination Awareness and Health Literacy

The population groups studied in this review were often unable to find, understand or use vaccine information and services [18,53,114]. Language was a significant barrier to accessing credible vaccine information [18], which was often related to the respective education level of the individual [18,72,84,85,111]. For instance, a study reported that an increased level of education was associated with higher VCRs with regard to rubella immunisation, especially among immigrant women [84]. Additionally, another study in Tempelhof and Neukölln refugee camps in Germany with parents who reported their children’s fully immunised status from memory (<5 years (28%), ≥5 years (74%), *p*  =  0.005), showed that these parents had more years of education on average with *p*-values of <0.05 [85]. Although not statistically significant, in contrast, one study reported that people with elementary to high school education level were less inclined to accept influenza vaccination [80].

Some studies indicated that vaccination advice had been provided through publications issued by individual and government health agencies [86,106,111]. However, vaccine literacy was unclear, and a key associated factor was interaction and familiarity with healthcare professionals [18,46,80,114,115]. For instance, Polish migrants in the UK were more likely to accept vaccine recommendations from healthcare providers in their resident country (86.7%) [73]. An interventional study in Sweden offering home visits aimed at supporting new parents in disadvantaged areas and improving parental efficacy (including vaccination knowledge, benefits and drawbacks) reported VCRs for MMR increased in children who had received six home visits [82]. In the same study, higher vaccine uptake correlated well with fewer inpatient episodes and emergency room visits [82].

Most studies lacked information on the details of recommendations provided to the study populations, as well as available vaccine information and messages. Unless vaccine information was provided in translated and simple, easy-to-understand formats, individuals sought information from family and friends. In these instances, lay-level information influenced decision making beyond the education level of the individuals [18]. Amongst disadvantaged groups in Romania, those with the lowest levels of education were least likely to think that the health facility provided quality services compared to comparable groups with higher education levels [87]. Some individuals were under the impression that knowledge gained from past experiences, such as immunisation of previous children or older siblings, gave them expertise and knowledge equivalent to professional training [21,114]. A study conducted in Greece and The Netherlands reported that asylum seekers emphasised the importance of educational activities for improving vaccine knowledge, such as seminars/presentations within the hosting facilities (55 of 61 participants) [47]. One approach used to increase vaccination awareness in the community was shown in the study where door-to-door household survey data collection staff also informed refugees of planned vaccinations and their importance and benefits to children [62].

#### 3.3.3. Barriers

Overall, most barriers were found at the healthcare service, community and individual levels. Significant barriers identified included insufficient cultural sensitivity, vaccine shortage, lack of trust in health services and vaccine safety, language barriers, discrimination, religious and cultural concerns, difficulty accessing vaccination follow-up, financial payments (where out-of-pocket expenditure is required), low-risk perception and dose incompletion. These mainly were linked to poor health literacy, lack of awareness of individual risks and lack of vaccine knowledge, which was particularly highlighted in COVID-19 vaccine studies. Despite this, concrete recommendations related to bridging the knowledge gap and health literacy for disadvantaged groups were lacking in most studies.

Strong indications were observed for the link between risk perception of adverse effects, disease susceptibility and community influence, as well as parental attitudes on the one hand and vaccine uptake on the other. Reasons against vaccination included the misperception that vaccination was unnecessary or not useful and disagreement with immunisation due to sociopolitical or religious belief issues [53,68,72,109]. There was also a lack of trust in and access to health services, unclear vaccine recommendations by healthcare professionals [24,27,65,72,107] and the quest for reassurance from people back in the respective home country or local community [21,68,109]. The level of deprivation (particularly for COVID-19 vaccines), including poverty, inadequate housing and low literacy in the arrival country for people on the move, also impacted vaccination uptake [18,46,95]. In addition, especially for COVID-19 vaccination, residential segregation aimed to reduce the spread of COVID-19 was also a limiting factor [42]. Overall, barriers to adult vaccinations were often an issue with the timing and location of appointments [76].

#### 3.3.4. Facilitators

There was evidence of improved knowledge about vaccines having the potential to increase vaccine uptake [53]. For migrants, the point of entry and holding level in the arrival country were considered optimal timing and settings for vaccination [47,59]. Having pragmatic approaches such as clear policy, national programmes and using educational institutions, such as schools, to administer vaccines, were considered a positive factor for increased vaccine coverage [73,74,109,113]. Training for healthcare workers and getting recommendations from professional staff, including community-based nurses and doctors, motivated vaccination uptake and, thus, fostered confidence [20,46,53,64,74,77,103,111]. In addition, having flexible appointments, easy planning and reminders were considered facilitators, as these could reduce the volume of incomplete vaccinations [53,72,109]. Bell et al. (2020) further highlighted that face-to-face communication was considered a much more effective approach to reaching communities and gaining their trust, using outreach strategies, under the “activation” [18].

At the healthcare level, leveraging the current public health workforce and increasing collaborative health education activities with local NGOs improved vaccination uptake among refugees [20,61,76]. At the community level, community involvement, religious support and spiritual endorsements were considered essential [53,109,112,116]. At the individual level, awareness of personal risk factors, access to health insurance or cost-free services and community vaccination centres were vital to encouraging vaccine uptake [18,42,53,66,109]. Generally, the decision to vaccinate was primarily made by mothers [23], and the most influential source of parental decisions to vaccinate their children was the healthcare staff, such as nurses and doctors, and, to a lesser extent, information from family and friends, the Ministry of Health and the internet [109,111]. Face-to-face communication was considered a more effective approach to reaching communities and gaining their trust, using community vaccine promotion outreach strategies [18]. Overall, previous vaccination experience significantly influenced subsequent vaccination decision making [73].

## 4. Discussion

The review reveals recent vaccination information on some disadvantaged communities, as distinct groups in most European countries are either unavailable or unpublished, with the identified studies representing only 19 of the 53 countries in the WHO European Region. The studies aimed to assess the prevention of 22 different infectious diseases, including vaccines against single diseases and combination vaccines against multiple conditions. The vaccine with the highest coverage rates was MMR, and for measles in particular, while vaccines requiring multiple doses had very low uptake because of missed return appointments for subsequent doses and poor follow-up in general. Vaccination coverage and uptake also differed by country of origin and certain demographic characteristics, especially among Middle Eastern and Sub-Saharan African migrants. Although large proportions of the populations were adults, most of the studies did not specify the exact vaccines assessed for adult groups. Furthermore, various factors, including trust in the health system, cultural factors and level of vaccine awareness, influenced the availability and uptake consideration of vaccination at different levels. The main barriers were at the healthcare service, community and individual level, while some facilitators, such as the need for increased vaccine knowledge, were identified at all levels. In addition, several drivers that influence vaccination decision making were identified, for example, accessibility and flexibility of vaccination services. The most common external driver, especially for COVID-19 vaccine uptake, was level of deprivation.

As shown in the review by Thomson et al. (2016), determinants of vaccine uptake can be grouped under five dimensions: access, affordability, awareness, acceptance and activation [117]. Within these dimensions are psychological, social and contextual factors associated with the different population groups. Context determinants include socioeconomic status, education level and health systems operations [118], which align with our findings. Understanding how the different determinants and dimensions influence vaccine coverage among disadvantaged groups is fundamental to identifying ways to improve vaccine uptake. This review shows that migrants are at risk for lower VCRs in their home country than in their countries of residence. Unless vaccination is included at the site of entry or soon after that, and unless the arrival country has achieved VCR levels consistent with herd immunity, there may be an increased risk of re-emergence or new outbreaks [4,5]. Therefore, it is vital to consider the country of origin of migrants and the known VCR there and to undertake efforts to match national coverage towards ensuring herd immunity. However, evaluating VCRs among countries in the WHO European Region is challenging due to migrants moving across countries, and VCRs being often influenced by what happens in border countries [119].

Most evidence in the included studies was based on self-reports from the populations who had been vaccinated, but recall of vaccinations received in the past was as low as 39% [47]. This observation is in line with work previously published by the Vienna Vaccine Safety Initiative [120]. Lack of knowledge of one’s own immunisation status may contribute to false reporting, which in turn increases the risk of VPD or, reversely, unnecessary duplicate vaccination, which is not cost-effective. As identified in other studies, having standardised digital vaccination records and/or databases that are frequently updated and widely accessible (for vaccine recipients and the providers they wish to share the data with) would improve follow-up and reduce gaps in vaccination coverage [4,121]. Additionally, developing online registries and cooperation between countries could allow for keeping track of administered vaccines in order to appropriately plan immunisation series and avoid unnecessary vaccinations [119]. Some progress has been made regarding digital records of COVID-19 vaccination in many European countries, but routine childhood vaccines and life course immunisations have been delayed or skipped during the pandemic [122]. Lastly, it is important to consider that vaccine uptake does not imply full protection or guarantee immunity. In particular, for individuals who did not complete the entire vaccine series in cases of vaccines requiring multiple doses and/or boosters, laboratory testing for vaccine protection may be required. Despite its shortcomings, serology testing provides the most accepted surrogate marker for vaccine protection [123,124]. Further improvements need to be made with regard to correlates of protection [125]. In this context, it may also be relevant to verify adequate protection in individuals who, although previously vaccinated, remain at risk of infection due to low antibody response, as is sometimes the case with the HBV vaccine. Looking further into individual variability, including immunological and sociodemographic factors, may help to tailor immunisation programs to the needs of specific vulnerable population groups. Other significant factors are risk awareness and acceptance of state authority, which may be influenced by religion, ethnicity and vaccine literacy. The lack of knowledge about adult vaccine recommendations and benefits and lack of funding have also been identified as essential factors to consider [126]. Lack of awareness regarding the importance of vaccines was commonly reported; however, the extent to which this affected vaccine uptake decision was unclear, even for COVID-19 vaccinations. The lack of clarity could be attributed to the complex intersectional relationship between vaccine uptake, individual factors (e.g., ethnicity and religion) and external factors (e.g., level of deprivation and location) [127]. Additionally, little is known about how the specific contents of vaccine communication or messages impact personal decisions and VCR. However, health literacy has been shown to predict vaccine acceptance regardless of country, age and type of vaccine [128]. To increase vaccine literacy, communication barriers need to be removed by providing translation services and cultural competence training (i.e., knowledge and skills to provide effective care for particular groups) [129]. Considering the interplay between the identified factors among disadvantaged and isolated communities and their role, it is crucial to consider all of the above when increasing vaccine uptake.

Of the five dimensions and determinants, affordability was not a frequently reported concern when vaccines were provided free of charge. However, the “activation” dimension, i.e., encouraging individuals to get vaccinated [117], was weak when barriers were identified, primarily cultural and communication-related factors, and when there was a lack of consistent and clear vaccine recommendations by healthcare providers. Identified drivers of vaccination showed that different factors, such as vaccine provider, economic status, deprivation and vaccine literacy, influenced uptake at different levels, from the national (wider population) to individual levels [128,130]. To bridge the gap between the different levels, especially among migrants, health literacy and language barriers need to be addressed through an integrated healthcare system [131,132]. This finding corroborates previous reports that have shown knowledge about vaccines, social influences and trust in the healthcare profession are strongly associated with vaccine uptake [3,13,133,134]. Addressing these elements requires direct engagement with local communities based on their unique contextual issues [135]. Different strategies, which utilise positive vaccination facilitators and removal of barriers, help to improve vaccination coverage. For instance, the three factors identified by Stein-Zamir et al. (2017), i.e., sociopsychological, health services-related and vaccine-perception-related, are crucial elements for any vaccination campaign [53]. As with religious beliefs, cultural factors play an important role in decisions to get vaccinated. Cultural sensitivities, stigma and the importance of peer influence in vaccine decision making are factors that will need to be taken into consideration when developing vaccine campaigns among disadvantaged groups, specifically migrants and refugees. Therefore, strengthening immunisation policies, surveillance and services for each identified group would be beneficial. However, vaccination policies and approaches sometimes differed across countries and government administration levels [9,136]. For instance, in the policy analysis by Ravensbergen et al. (2019), only 6 of 32 countries assessed had comprehensive policies specific to the vaccination of migrants, while 19 countries applied their national vaccination schedule to migrant vaccinations [9].

Possible strategies to implement in vaccination policy and programs as identified from this review include point-of-entry disease screening with matching vaccination supply for new migrants; establishing vaccine policies to be enforced at healthcare centres would provide reminders for isolated communities, including other institutions in vaccine planning such as schools, health insurance providers and traveller centres. Preferred vaccination authorities such as NGOs and public healthcare systems that utilise community-based nurses could also be considered. In addition, providing information in ways that portray cultural and religious considerations could encourage higher vaccination uptake. Community and individual barriers could be addressed through improved advocacy actions that involve leaders of the identified communities in vaccine planning, clear messaging that outlines vaccination details, which include free services (where applicable), reducing hesitancy by addressing personal concerns such as side effects and complications and enhancing awareness of transmission risks, especially among children. On the other hand, at the health service level, reducing barriers such as waiting time and obtaining an appointment and addressing vaccine shortages would enhance the quality and timeliness of vaccination services while motivating vaccination uptake. Improved healthcare service can be achieved by implementing local hubs that target and cover smaller regions or specific groups. For consistency of services, digital tools may support scheduling, provision and monitoring of vaccination information while increasing vaccine communication and uptake. This approach would strengthen the “activation” dimension of the determinants of health, which focuses on encouraging individuals towards vaccination uptake [117].

Overall, the data on VCRs show that the continued focus and prioritisation of paediatric immunisation may not automatically translate into improved uptake of life-course or adult vaccinations [137]. Key factors to improve VCR among underserved populations include education and improved vaccine delivery [7,137,138] However, this review showed that a one-size-fits-all approach might not be successful in diverse population groups where social determinants of health need to be considered. Furthermore, more qualitative research regarding vaccine coverage information, which would provide more insights and perceptions related to vaccine decision making, could provide insight into experiential factors and perspectives, highlighting emotional and psychological factors underlying vaccine decision making. The European Vaccine Action Plan explicitly identified goals for sustaining a polio-free status, elimination of measles and rubella, control of HBV infection and meeting 95% coverage of three doses of DTP-containing vaccines [7]. However, shared vaccination metrics and outcome measures for vaccine performance are not available nor monitored in all European countries and may vary from country to country or may vary within the same country [10]. Vaccine funding and delivery are the responsibility of individual countries in Europe [10], resulting in inconsistent national and regional approaches, which affect cross-border populations, in particular [10]. Greater alignment can be achieved through moving vaccination services closer to the identified disadvantaged, isolated and hard-to-reach community groups, while using consistent methodologies for the monitoring of the performance of local services, including digital tools that would improve data quality and consistency. This is fundamental, especially now as we are faced with an increased movement of populations across Europe, underlining the ongoing need for flexibility, resilience and crisis readiness of vaccination services. Also, medical and public health services need to adapt their responses to the needs of isolated, disadvantaged and difficult-to-reach population groups to reduce disease outbreaks which affect vulnerable populations disproportionally. Summary recommendations from the review are shown in Text Box 1.

Box 1 **Summary of review recommendations**

Interventions specific to target communities are key to eliminating and preventing vaccine-preventable disease outbreaks. Since prevention and control in disadvantaged, isolated and difficult-to-reach communities are not efficient using top-down healthcare models alone, tailored community-based interventions need to be deployed. As such, interventions to reduce gaps in VCRs in non- and under-vaccinated populations need to be adapted to the specific population.

To increase vaccination uptake among these populations, we recommend consideration of the following:

- Identification of strategies for effectively reaching isolated populations with information about vaccinations;
- Providing early screenings, testing and immediate vaccination for migrant and traveller communities;
- Improving health monitoring by establishing secure databases, implementing vaccination policies that take into consideration the unique factors influencing specific population groups;
- Improving knowledge and understanding of vaccines by providing more information about personal benefits and risks;
- Using new communication techniques (social media) in the active and effective reaching of isolated groups with vaccine information establishing secure immunisation databases that can be frequently updated;
- Supporting vaccine literacy by providing more information to both health providers and isolated, disadvantaged and difficult-to-reach population groups;
- Enhancing community intervention strategies and involvement;
- Strengthening advocacy with community leaders and representatives.

### Limitations

The review was limited to peer-reviewed English-language publications only; hence, grey literature was not included. Therefore, the data may not reflect within-country variations, and their availability may be subject to publication bias. Additionally, strict inclusion criteria focusing on quantitative data may have led to the exclusion of relevant qualitative studies. Grey literature that may have had relevant and interesting information was also excluded due to the likely high volume of data from all included countries, report overlaps and evidence inconsistency. In addition, although the review search was updated, the data available at the time of the revised search may have changed, especially with the administration of COVID-19 vaccines and the publication of studies, which likely impact the differences and timeliness of publicly available data across different countries. Additionally, wide variations in study aims, intervention approach and countries reported made it challenging to perform extensive comparisons and in-depth statistical analysis. Future country-specific studies may augment this review with national datasets.

A key strength of this review is that it looked at a wide range of vaccine-preventable diseases among various disadvantaged groups in all 53 countries of the WHO European Region, providing a broader assessment beyond the European Union (EU) region itself. Our findings were in line with other key publications while highlighting significant gaps and deficiencies in the current body of evidence. More qualitative and quantitative research is required in different population groups to understand how the deployment of effective vaccine strategies or campaigns may be tailored to improve VCR among isolated, disadvantaged and difficult-to-reach populations. Additionally, more innovative and improved immunisation services need to be developed for these populations in Europe and beyond.

## 5. Conclusions

Successful vaccine uptake depends upon national policies and supplies, health professionals offering it to individuals and the willingness of the specific population group to participate in an immunisation program. To develop interventions that effectively increase vaccination rates among disadvantaged, isolated and difficult-to-reach communities, it is necessary to understand current VCRs and factors that affect uptake, both of which are influenced by several contextual factors. Our results indicate that, to improve vaccine uptake, interventions should not be limited to healthcare services, and different societal groups need to be engaged actively while reducing specific barriers to access that these groups may be facing. This review shows that intervention focused on improving vaccine policies within and across different countries, providing knowledge related to vaccines, involvement of user-centred and privacy-preserving digital tools and effective VPD surveillance systems could help allocate resources to where they are most needed to improve vaccine uptake. More qualitative and quantitative research is required in different isolated, disadvantaged and difficult-to-reach population groups to understand how the deployment of different vaccine strategies or campaigns may be tailored to specific groups.

## Figures and Tables

**Table 1:** Summary of vaccines identified in the included studies.

Vaccine Types	Countries Represented	No. of Studies	Included Studies (Authors, Year)
BCG	England, Germany, Scotland, Switzerland, Turkey	7	Bell et al. (2020) [18], Boukamel et al. (2020) [19], Ergönül et al. (2019) [20], Gorman et al. (2019) [21], Mueller-Hermelink et al. (2018) [22], Öztaş et al. (2020) [23], Ergönül et al. (2019) [20]
COVID-19	Israel, Italy, Norway the United Kingdom, Wales	19	Ali-Saleh et al. (2022) [24], Bentivegna et al. (2022) [25], Blakeway et al. (2022) [26], Cook et al. (2022) [27], Gaughan et al. (2022) [28], Glampson et al. (2021) [29], Gorelik et al. (2022) [30], Hall et al. (2021) [31], Iacoella et al. (2021) [32], Kraft et al. (2022) [33], Martin et al. (2021) [34], Muhsen et al. (2021) [35], Nafilyan et al. (2021) [36], Nguyen et al. (2022) [37], Perry et al. (2021) [38], Saban et al. (2021) [39], Taubman- Ben-Ari et al. (2022) [40], Tessier et al. (2022) [41], Watksinson et al. (2022) [42]
Diphtheria (DTP/DaPT/DTap)	Denmark, England, Finland, Germany, Greece, Israel, Italy, The Netherlands, Switzerland, Turkey, the United Kingdom, Wales	14	Dixon et al. (2016) [43], Ergönül et al. (2019) [20], Freidl et al. (2018) [44], Jablonka et al. (2017) [45], Jackson et al. (2017) [46], Louka et al. (2019) [47], Mellou et al. (2019) [48], Nakken et al. (2018) [49], Perry et al. (2020) [50], Sane et al. (2016) [51], Staehelin et al. (2019) [52], Stein-Zamir et al. (2017) [53], Stein-Zamir et al. (2019) [54], Vita et al. (2019) [55]
Hepatitis (A, B, C)	England, Finland, Germany, Greece, Israel, Italy, The Netherlands, Spain, Switzerland, Turkey	21	Cuomo et al. (2019) [56], Ergönül et al. (2019) [20], Fougère et al. (2018) [57], Freidl et al. (2018) [44], Führer et al. (2016) [58], Iacoella et al. (2021) [32], Jablonka et al. (2017) [45], Karaşahin et al. (2021) [59], Köse et al. (2017) [60], Louka et al. (2019) [47], Mazzitelli et al. (2021) [61], Mellou et al. (2017) [62], Mellou et al. (2019) [48], Norman et al. (2021) [63], Öztaş et al. (2020) [23], Serre-Delcor et al. (2018) [64], Staehelin et al. (2019) [52], Stein-Zamir et al. (2019) [54], Taylor et al. (2019) [65], Vita et al. (2019) [55], Vu et al. (2020) [66]
HiB	Denmark, England, Greece, Israel, Turkey	5	Dixon et al. (2016) [43], Ergönül et al. (2019) [20], Mellou et al. (2019) [48], Nakken et al. (2018) [49], Stein-Zamir et al. (2019) [54]
Influenza	England, Germany, Greece, Israel, Italy, The Netherlands, Scotland, Turkey, the United Kingdom, Wales	20	Bechini et al. (2018) [67], Bell et al. (2020) [18], Bielecki et al. (2019) [68], Bielecki et al. (2020) [69], Boddington et al. (2019) [70], Fortunato et al. (2018) [71], Glatman-Freedman et al. (2019) [72], Gorman et al. (2019) [21], Gorman et al. (2020) [73], Hardelid et al. (2016) [74], Hungerford et al. (2018) [75], Iacoella et al. (2021) [32], Jackson et al. (2017) [46], Loiacono et al. (2020) [76], Louka et al. (2019) [47], Natan et al. (2016) [77], Perniciaro et al. (2018) [78], Shahbabi et al. (2021) [79], Watksinson et al. (2022) [42], Yakut et al. (2020) [80]
Measles, Mumps, Rubella (MMR/MMRV/MMRV1/ MMRV2)	Denmark, England, Germany, Greece, Israel, Italy, The Netherlands, Scotland, Slovakia, Spain, Sweden, Switzerland, Turkey, Wales	29	Bell et al. (2020) [18], Brockmann et al. (2016) [81], Burström et al. (2020) [82], Ceccarelli et al. (2018) [83], Dixon et al. (2016) [43], Ergönül et al. (2019) [20], Fabiani et al. (2017) [84], Freidl et al. (2018) [44], Fozouni et al. (2019) [85], Georgakopoulou et al. (2018) [86], Habersaat et al. (2020) [87], Hagstam et al. (2019) [88], Haider et al. (2019) [89], Hudečková et al. (2020) [90], Jablonka et al. (2017) [45], Jablonka et al. II (2017) [91], Louka et al. (2019) [47], Mellou et al. (2019) [48], Nakken et al. (2018) [49], Norman et al. (2021) [63], Öztaş et al. (2020) [23], Perry et al. (2020) [50], Staehelin et al. (2019) [52], Stein-Zamir et al. (2017) [53], Stein-Zamir et al. (2019) [54], Suppli et al. (2018) [92], Van Den Heuvel R. et al. (2018) [93], Vita et al. (2019) [55], Werber et al. (2017) [94]
Meningococcal/MCV/MenC	England, Greece, Italy, Wales	4	Dixon et al. (2016) [43], Georgakopoulou et al. (2018) [86], Perry et al. (2020) [50], Vita et al. (2019) [55]
Pertussis	England, Italy, Turkey, Wales	5	Byrne et al. (2017) [95], Dixon et al. (2016) [43], Perry et al. II (2020) [96], Vita et al. (2019) [55], Yakut et al. (2020) [80]
Pneumonia/PCV/Invasive pneumococcal disease (IPD)/Prevnar/CPV	Denmark, England, Greece, Israel, Italy, Turkey	6	Dixon et al. (2016) [43], Ergönül et al. (2019) [20], Mellou et al. (2019) [48], Nakken et al. (2018) [49], Öztaş et al. (2020) [23], Stein-Zamir et al. (2019) [54], Vita et al. (2019) [55]
Polio/OPV/IPV	Denmark, England, Germany, Greece, Israel, Italy, The Netherlands, Turkey, the United Kingdom	13	Dixon et al. (2016) [43], Fozouni et al. (2019) [85], Freidl et al. (2018) [44], Hvass et al. (2019) [97], Jackson et al. (2017) [46], Louka et al. (2019) [47], Mellou et al. (2019) [48], Nakken et al. (2018) [49], Öztaş et al. (2020) [23], Stein-Zamir et al. (2019) [54], Tayfur et al. (2019) [98], Veronesi et al. (2019) [99], Vita et al. (2019) [55]
Rotavirus	England/the United Kingdom	3	Byrne et al. (2017) [95], Dixon et al. (2016) [43], Hungerford et al. II (2018) [100]
Shingles	The United Kingdom	1	Ward et al. (2017) [101]
Tetanus	England, Germany, Greece, Italy The Netherlands, Switzerland, Turkey, the United Kingdom	11	Affani et al. (2020) [102], Dixon et al. (2016) [43], Ergönül et al. (2019) [20], Fozouni et al. (2019) [85], Freidl et al. (2018) [44], Führer et al. (2016) [58], Jablonka et al. (2017) [45], Jackson et al. (2017) [46], Louka et al. (2019) [47], Staehelin et al. (2019) [52], Vita et al. (2019) [55]
Tuberculosis	Denmark, Italy, Switzerland	3	Ahmad et al. (2020) [103], Fritschi et al. (2021) [104], Iacoella et al. (2021) [32]
Varicella/VZV	Germany, The Netherlands Spain, Switzerland, Turkey, the United Kingdom	7	Ergönül et al. (2019) [20], Freidl et al. (2018) [44], Jablonka et al. II (2017) [91], Norman et al. (2021) [63], Öztaş et al. (2020) [23], Staehelin et al. (2019) [52], Ward et al. (2017) [101]
Whooping cough	The United Kingdom	1	Jackson et al. (2017) [46]
General/Multiple vaccines/Combination vaccines	Belgium, Denmark, Italy, Poland, Switzerland, Turkey	6	Dam Larsen et al. (2017) [105], Decuyper et al. (2019) [106], Ganczak et al. (2021) [107], Pohl et al. (2017) [108], Vita et al. (2019) [55], Öztaş et al. (2020) [23]

NOTE: Details of each study are presented in Supplementary File S2.

**Table 2:** Summary of identified drivers, barriers and facilitators of vaccination.

Levels	Drivers	Barriers	Facilitators
National	Official authorities NGOs National Healthcare Employees Social media Economic status Deprivation index	Insufficient cultural sensitivity Vaccine shortage Pre-existing social inequalities	Point of entry and holding level in Europe as optimal timing for vaccination Nationwide vaccine programme Policy of mandatory vaccine Non-mandatory system option Television ads Health surveillance system
Healthcare service	Public healthcare facilities Professional healthcare staff Immunisation database	Finances where payments were required Lack of trust in health services, health approaches and need for opinions from home country Poor access to basic facilities at clinics Long waits Overload and stressful environment at clinic Follow-up challenges (mostly refugees)	Free-of-charge preventive health service Reminders from clinic, schools, HMO on upcoming scheduled visits Flexible appointments, easy planning Healthcare staff education and training Recommendation from healthcare staff Health education collaboration with local NGOs Pre-existing condition monitoring
Community	Social contacts (information from family and friends) Community and religious leaders Displacement camp residence	Deprivation Discrimination Religious and cultural concerns Number of cultural mediators Refugee camp population changes and closures Negative and scientifically “incorrect” opinions Negative peer pressure Poverty Residential segregation (especially COVID-19)	Promotional and outreach programmes Increased educational activities and resources, including school-led events Community involvement, religious support and spiritual endorsements Obligation to the community Familial support network Camp dwelling Importance of preventing diseases and protecting the health of children
Individual	Parental decisions (mostly mothers) Face-to-face communication Finance Previous experience Personal documentation	Low-risk perception Lack of faith in vaccine’s need, safety Fear of side effects and complications Never being offered vaccination Delayed receipt of the first dose Not returning after their initial dose Transportation challenges Language barrier and low literacy Undocumented status Short residence duration Certain demographic factors (e.g., being female, high birth order, ethnicity, most deprived locations)	Having risk factors Awareness and understanding that unvaccinated children pose risk of transmissible infection to others Lower-income predicted higher compliance Health insurance Ability of individuals to be reached by, or to reach, recommended vaccines School attendance Work employment Information material in own language Certain demographic factors (e.g., age of the index child, vaccination status of other family members, education)

## Data Availability

Not applicable.

## Supplementary Materials

The following supporting information can be downloaded at: https://www.mdpi.com/article/10.3390/vaccines10071038/s1. File S1: sample search strategy; File S2: studies included in review showing vaccination uptake among different populations and countries (non-COVID-19 vaccines); File S3: studies included in review showing vaccination uptake among different populations and countries (COVID-19 vaccine only).
