# Supplementary material for: Access to Vaccination among Disadvantaged, Isolated and Difficult-to-Reach Communities in the WHO European Region: A Systematic Review
Source: Vaccines (Basel). 2022 Jun 28;10(7):1038. doi: 10.3390/vaccines10071038 (PMC9324407; doi:10.3390/vaccines10071038)
Supplement: Supplementary file 1 [file vaccines-10-01038-s001.zip › Supplementary S1- OVID MEDLINE Search strategy.pdf]

**Supplementary S1: OVID MEDLINE Search strategy.**

*Example of first search conducted in November 2020. The same terms were used in the updated search in March 2022*

| # ▲ | Searches |
|---|---|
| 1 | exp Vaccines/ |
| 2 | exp Vaccination/ |
| 3 | exp Immunization/ |
| 4 | exp Immunization Programs/ |
| 5 | (vaccin" or immunis" or immuniz").mp. |
| 6 | 1 or 2 or 3 or 4 or 5 |
| 7 | exp Vulnerable populations/ |
| 8 | exp Ethnic Groups/ |
| 9 | exp "Transients and Migrants"/ |
| 10 | exp Refugees/ |
| 11 | (disadvantage" or vulnerable" or "ethnic minorit"" or migrant" or refugee" or asylum" or nomad" or squatter" or transient" or "risk group"" or traveller" or gyps" or socioeconomic" or "socio economic"").mp. |
| 12 | ("non eu" or "non-europe"").mp. |
| 13 | ((visit" or guest") adj5 (work or employment or job or jobs)).mp. |
| 14 | 7 or 8 or 9 or 10 or 11 or 12 or 13 |
| 15 | exp Europe/ |
| 16 | exp Albania/ |
| 17 | exp Andorra/ |
| 18 | exp Armenia/ |
| 19 | exp Austria/ |
| 20 | exp Azerbaijan/ |
| 21 | exp Belarus/ |
| 22 | exp Belgium/ |
| 23 | (Bosnia and Herzegovina).mp. [mp=title, abstract, original title, name of substance word, subject heading word, floating sub-heading word, keyword heading word, organism supplementary concept word, protocol supplementary concept word, rare disease supplementary concept word, unique identifier, synonyms] |
| 24 | exp Bulgaria/ |
| 25 | exp Croatia/ |
| 26 | exp Cyprus/ |
| 27 | exp Czech Republic/ |
| 28 | exp Denmark/ |
| 29 | exp Estonia/ |
| 30 | exp Finland/ |
| 31 | exp France/ |
| 32 | exp "Georgia (republic)"/ |
| 33 | exp Germany/ |

| | |
|---|---|
| 34 | exp Greece/ |
| 35 | exp Hungary/ |
| 36 | exp Iceland/ |
| 37 | exp Ireland/ |
| 38 | exp Israel/ |
| 39 | exp Italy/ |
| 40 | exp Kazakhstan/ |
| 41 | exp Kyrgyzstan/ |
| 42 | exp Latvia/ |
| 43 | exp Lithuania/ |
| 44 | exp Luxembourg/ |
| 45 | exp Malta/ |
| 46 | exp Monaco/ |
| 47 | exp Montenegro/ |
| 48 | exp Netherlands/ |
| 49 | exp "Republic of North Macedonia"/ |
| 50 | exp Norway/ |
| 51 | exp Poland/ |
| 52 | exp Portugal/ |
| 53 | exp Moldova/ |
| 54 | exp Romania/ |
| 55 | exp Russia/ |
| 56 | exp San Marino/ |
| 57 | exp Serbia/ |
| 58 | exp Slovakia/ |
| 59 | exp Slovenia/ |
| 60 | exp Spain/ |
| 61 | exp Sweden/ |
| 62 | exp Switzerland/ |
| 63 | exp Tajikistan/ |
| 64 | Turkey.mp. |
| 65 | exp Turkmenistan/ |
| 66 | exp Ukraine/ |
| 67 | ("United Kingdom" or UK or "Great Britain" or Britain or "Northern Ireland" or England or Scotland or Wales).mp. |
| 68 | exp Uzbekistan/ |
| 69 | 15 or 16 or 17 or 18 or 19 or 20 or 21 or 22 or 23 or 24 or 25 or 26 or 27 or 28 or 29 or 30 or 31 or 32 or 33 or 34 or 35 or 36 or 37 or 38 or 39 or 40 or 41 or 42 or 43 or 44 or 45 or 46 or 47 or 48 or 49 or 50 or 51 or 52 or 53 or 54 or 55 or 56 or 57 or 58 or 59 or 60 or 61 or 62 or 63 or 64 or 65 or 66 or 67 or 68 |
| 70 | 6 and 14 and 69 |
| 71 | limit 70 to yr="2015 - 2020" |
