# Supplementary material for: Access to Vaccination among Disadvantaged, Isolated and Difficult-to-Reach Communities in the WHO European Region: A Systematic Review
Source: Vaccines (Basel). 2022 Jun 28;10(7):1038. doi: 10.3390/vaccines10071038 (PMC9324407; doi:10.3390/vaccines10071038)
Supplement: Supplementary file 1 [file vaccines-10-01038-s001.zip › Supplementary S2 - Included non-COVID studies.pdf]

Supplementary File S2

Table A: Studies included in review showing vaccination uptake among different populations and countries (non-COVID-19 vaccines) (n=87)

	Author and year of study	Country(s)	Vaccine/ Disease reported	Study design and period of data collection	Study population, comparison group and sample size	Vaccine coverage/Uptake	Quality range
1.	Affani et al. (2020)[1]	Italy	Tetanus	Cross-sectional (Prevalence) (Serology) 2004-2019	Migrants/Refugees (Recent arrivals)<br>- All ages (n=2,150)	**Sample (n=2,150):** AFR (49.4%, n=1,063), EMR (13.4%, n=289), EUR (10.8%, n=232), SEAR (10.2%, n=219), AMR (8.7%, n=186), WPR (7.5%, n=161).<br><br>- 22.3% (480/2,150) had no protective antibodies (<0.10 IU / mL),<br>- 45.2% (971/2,150) intermediate range (0.11-1.00 IU/mL)<br>- 32.5% (699/2,150) high titer of antibodies (> 1.00 IU/mL).<br><br>**-- Protective titer (>1.00 IU/mL) by region (n=699):** WPR (23.0%), AFR (27.2%), EMR (27.0%), AMR (42.5%), SEAR (49,3%), EUR (46.6%)<br>**--Protective antibodies:** AFR (28.2%), EMR (28.0%).	Medium
2.	Ahmad et al. (2020)[2]	Denmark	Tuberculosis	Cross-sectional (Prevalence) 2017-2018	Refugee<br>- Children <16 years (n=244)	**Valid BCG testing results (n=173)**: 51.6 % (126/244) vaccinated, 19.6% (47/244) not vaccinated/unknown	Low
3.	Bechini et al. (2018)[3]	Italy	Influenza	Prevalence 2015-2017	Disadvantaged areas (Deprivation)<br>- Adults ≥ 65 years (n="not reported")	**Vaccine coverage 2015/16 and 2016/17 combined**: 54.7%<br>- **2015/16**: 57.3%<br>- **2016/17**: 53.8%	Medium
4.	Bell et al. (2020)[4]	England	BCG<br>Influenza<br>MMR	Qualitative (Semi-structured interviews) 2017-2018	Migrants (Romania and Roma)<br>- Mothers (n=9) representing Children 0.5-21 years (n=28)	Of n=28 (71.4%) children,<br>- 64.3% (18/28) children fully vaccinated<br>- 17.9% (5/28) BCG only<br>- 17.9% (5/28) unvaccinated	Medium
5.	Bielecki et al. (2019)[5]	Scotland	Influenza	Cohort (Prospective, Serology) 2015-2017	Migrants (Polish)<br>- Children 5-12 years (n=387)<br><br>Other children 5-12 years (n=535)<br>- White British<br>- Other identified ethnic minorities<br>- Unknown ethnicity	**Polish**: Year 2016 (22.2%, 86/387), Year 2017 (25%, 93/372)<br>- Difference (95% CI): 3.8% (–3.24–8.84%), p = 0.364 (NS)<br><br>**White British**: Year 2016 (62.6%, 186/297), Year 2017 (70.7%, 205/290)<br>- Difference (95% CI): 8.1% (0.47–15.73%), p = 0.038<br><br>**Other identified ethnic minorities**: Year 2016 (54.4%, 93/171), Year 2017 (60.9% 117/192)<br>- Difference (95% CI): 6.49% (–3.68–16.68%), p = 0.211	Medium

						**Unknown ethnicity**: Year 2016 (55.2%, 37/67), Year 2017 (42.9%, 27/63)<br>- Difference (95% CI): –12.3% (–4.9–29.5%), p = 0.161	
6.	Bielecki et al. (2020)[6]	Scotland	Influenza	Cohort<br>2016-2018	Migrants (Polish, Other ethnic minorities)<br>- Children (Polish n=404, Other ethnic minorities n=577)<br><br>Comparision<br>- UK (n=883)	**Influenza vaccine uptake rates**<br>By year and response (Immunised, Refused, Non-Returned questionnaire); (% Difference between 2017 & 2018 (95% CI) Significance)<br><br>**Polish (n= 404)**<br>**- Immunized:** Year 2016 (21.0%), Year 2017 (20.8%), Year 2018 (21.3%); 0.5% (−5.1–6.1%), p= 0.816<br>**- Refused:** Year 2016 (20.8%), Year 2017 (39.9%), Year 2018 (43.1%); 3.2% (−3.6–9.9%) p= 0.356<br>**- Non-Returned:** Year 2016 (45.3%), Year 2017 (39.4%), Year 2018 (35.6%); 3.8% (−2.8–10.5%), p= 0.265<br><br>**Other ethnic minorities (n=577)**<br>**- Immunized:** Year 2016 (46.1%), Year 2017 (49.9%), Year 2018 (53.9%); 4.0% (−1.7–9.7%)p= 0.174<br>**- Refused:** Year 2016 (5.7%), Year 2017 (10.9%), Year 2018 (19.1%); 8.2% (4.1–12.3%)p< 0.0001<br>**- Non-Returned:** Year 2016 (36.6%), Year 2017 (39.2%), Year 2018 (27.0%); −12.2% (−6.7–17.6%)p< 0.0001<br><br>**UK (n=883)**<br>- **Immunized:** Year 2016 (49.3%), Year 2017 (58.0%), Year 2018 (60.7%); 2.7% (−1.9–7.3%), p= 0.248<br>- **Refused**: Year 2016 (4.5%), Year 2017 (7.5%), Year 2018 (9.7%); 2.2% (−0.4–4.8%), p= 0.099<br>- **Non-Returned:** Year 2016 (33.7%), Year 2017 (34.5%), Year 2018 (29.6%); −4.9% (−0.5–9.2%), p< 0.05	Low

7.	Boddington et al. (2019)[7]	UK	Influenza	Case-control<br>2015-2016	Migrants (Ethnic minorities)<br>- Children 2 to 16 years, general (n=977)	**Participants (n=977):** Vaccinated (30.4%, n=297), Not vaccinated (69.6%, n=680)<br><br>**Participant ethnicity distribution:** Asian (n=20), Black (n=69), White (n=129), Other (n=745), Missing data (n=14)<br><br>**By case/control groups and ethnicity group**<br>- **Vaccinated (n=297):** Cases (26.3%), Control (73.7%)<br>--- **Cases (n=78):** Asian (60%, 3/5), Black (25.8%, 8/31), White (24.6%, 14/57), Other (21.2%, 53/250)<br>--- **Controls (n=219):** Asian (33.3%, 5/15), Black (36.8%, 14/38), White (34.7%, 25/72), Other (35.2%, 174/495), Missing (11.1%, 1/9)<br><br>- **Not vaccinated (n=680):** Cases (39.7%), Control (60.3%)<br>--- **Cases (n=270):** Asian (40% 2/5), Black (74.2%, 23/31), White (75.4%, 43/57), Other (78.8, 197/250), Missing (100%, 5/5)<br>--- **Controls (n=410):** Asian (66.7%, 10/15), Black (63.2%, 24/38), White (65.3% 47/72), Other (64.8%, 321/495), Missing (88.9%, 8/9)	High
8.	Boukamel et al. (2020)[8]	Switzerland	**BCG**	Cohort<br>2015-2017	Newly arrived migrant<br>- Children, Adolescents 0-18 years (n=253)	28% (72/253) BCG vaccination<br><br>- 50% (6/11) false-positive tuberculin skin test<br>- 24% (37/152) negative tuberculin skin test vaccinated with BCG (p = 0.03)<br>- 6/43 (14%) BCG-vaccinated non-tuberculosis patients had false-positive tuberculin skin tests	High
9.	Brockmann, et al. (2016)[9]	Germany	Not specified	Cross-sectional (Prevalence)<br>2014-2015	Asylum Seekers<br>- All ages (n=2256)	28% (642/2256) received at least one vaccination<br>89% (571/2256) vaccinated as part of the newly developed vaccination concept<br>6% vaccinated in accommodations not previously included in the new concept<br>58% vaccination rate in the enclosed accommodations<br><br>Additional analysis of children who completed entire period of primary simmunisation (G4) for MMR, from 11th to 14th Month of life in the district<br>- 38% (8/21) children were vaccinated against MMR (95% CI [21–66%])<br>- 85% of 2-year-olds in the Reutlingen district were vaccinated	Medium

10.	Burström et al. (2020)[10]	Sweden	MMR	RCT<br>(Quasi-experiment)<br>2013-2016	Disadvantaged areas<br>- Children (n=261)<br><br>Home visit intervention (n=81) compared two control groups [Control (n=74) and Rinkeby (n=106)]	**Received MMR vaccine:** 95% CI [(Intervention vs Both control groups), (Control vs Rinkeby), p-value]<br><br>**0-36 months:** 95.1%, 94.6%, p= 0.019<br>**0-18 months:** 87.6%, 85.1%, p= 0.07<br>**18-36 months:** 95.1%, 94.6, p= 0.019	High
11.	Byrne et al. (2017)[11]	England	Pertussis<br>Rotavirus	Cross-sectional<br>- Maternal pertussis (2014-2015)<br>- Infant rotavirus (2014-2016)	Disadvantaged areas<br>- Adults (n=38,261 women)<br>- Children (n=91,578 infants)<br><br>National representation<br>- Adults (n=191,533 women)<br>- Children (n=459,074 infants)	**Coverage** % (n/N), **Adjusted coverage difference** compared with least deprived (difference, CI, p-value)<br><br>**Most deprived areas**<br>**- Pertussis (Mothers):** 49.4% (18,890/38,261), (−14·0, CI: −13·2 - −14·8, p= <0·001)<br>**- Rotavirus (Infants):** 83.7% (76 606/91,578), (−4.4 (CI: −4·7- −4·0, p= <0·001)<br><br>**National representation coverage**<br>**- Pertussis (Mothers):** 57.4% (109,927/191,533), CI 57.2 – 57.6<br>**- Rotavirus (Infants):** 86.7% (398,187/459,074), CI 86.6 – 86.8	Medium
12.	Ceccarelli et al. (2018)[12]	Italy	Measles,<br>Rubella	Cohort<br>2016	Asylum seekers (Eritrean, Malian, Gambian, Senegalese, Nigerian, Pakistan, Bangladesh)<br>- Adults 18 to 60 years (n=256)	**Prevalence of measles IgG antigen positivity**<br>- Eritrea (79.9%, 107/134), Nigeria (93.3%, 14/15), Gambia (2.6%, 19/23), Senegal (82.4%, 14/17), Mali (88%, 22/25), Pakistan (100%, 27/27), Bangladesh (100%, 15/15)<br><br>- Seroprevalence observed for Senegal, Mali, Nigeria, Pakistan and Bangladesh measles IgG greater than vaccinal coverage reported by WHO after 1 dose of vaccine	Low
13.	Cuomo et al. (2019)[13]	Italy	HBV	Cross-sectional<br>(Serology)<br>2016	Refugees, Asylum seekers, Economic migrants<br>- Adults (n=304)	5.6% (17/304) HBsAG vaccinated<br><br>- 12.2% (37/304) HBsAg positivity (CI 95% 0.08–0.16)<br>- 2.3% (7/304) HbcAb isolated positivity (CI 95% 0.01–0.04)<br>- 28.6% (87/304) Both HbcAb and HBsAb positivity as a previous HBV infection (CI 95% 0.23–0.33)<br>- 28.6% (87/304) HBsAg negative (CI 95% 0.23–0.33)<br>- 3.3% (10/304) HCVAb positivity (CI 95% 0.01–0.05)<br>- 10.2% (31/304) TB positive at Mantoux test (CI 95% 0.07–0.14)	High

14.	Dam Larson et al. (2017)[14]	Denmark	General	Cross-sectional (Prevalence) 2015	Travellers<br>- All ages (n=240)<br><br>Migrants (Eritrea, Romania, Somalia, Thailand, Congo)<br>- All ages (n=17)	289 diagnosed travel-related diseases<br>**10 potentially preventable by vaccines:** influenza A (n=2), typhoid fever (n=2), military/non-pulmonary tuberculosis (n=3), Varicella zoster(n=1),, hepatitis A(n=1), Pneumococcalmeningitis (n=1)<br><br>**All patients with vaccine-preventable diseases had unknown vaccination status,** except patients with one with typhoid fever (1=vaccinated) and tuberculosis (all from endemic countries, almost certainly BCG vaccinated)	Low
15.	Decuyper et al. (2019)[15]	Belgium	Routine and Non-routine vaccines	Cross-sectional (Data review) 2008-2016	Expatriate (Belgian)<br>- Children 0-18 years (n=116)	87% (140/161) met routine vaccination recommendations<br><br>- **Two doses HAV**: 9% (12/161)<br>- **Typhoid**: 21% (29/161)<br>- **Rabies full vaccination**: 8% (11/161)<br>- **Incomplete but with at least one routine vaccine**: 10% (16/161)<br><br>- **Yellow fever**: 61% (52/85)<br>- **Meningococcal ACW135Y**: 28.6% (20/70) (16 unconjugated, 4 conjugated)<br><br>- **BCG vaccine**: 84.7% (72/85) showed negative<br>- **TST**; 13.9% (10/72) received a BCG vaccine	Medium
16.	Dixon et al. (2016)[16]	United Kingdom (England)	Rotavirus, T/D/Po/Pe, MenC Hib, Pneumococcal MMR1 MMR2	Cross-sectional (Data review) 2015	Irish travelling community<br>- Children (n = 214)<br><br><br>Non-Travellers<br>- Children (n = 776)	**Travelling community**<br>**- Rotavirus**: 47.6% (10/21) covered<br>**- T/D/Po/Pe (2 months)**: 63.1% (135/214)<br>**- T/D/Po/Pe (3 months)**: 59.2% (126/213)<br>**- T/D/Po/Pe (4 months)**: 54.0% (114/211)<br>**- MenC**: 52.0% (104/200)<br>**- Hib**: 52.5% (105/200)<br>**- Pneumococcal**: 47.4% (55/116)<br>**- MMR1**: 54.0% (108/200)<br>**- MMR2**: 46.7% (86/184)<br>**- T/D/Po/Pe (B1)**: 45.7% (84/184)<br>**- T/D/Po/Pe (B2)**: 15.2% (7/46)<br>**- MenC (teenage B)**: 0% (0/32)<br>**- HPV**: 3.6% (1/28)<br><br>**Non-Travellers**<br>**- Rotavirus**: 92.2% (59/64)<br>**- T/D/Po/Pe (2 months)**: 95.7% (742/775)<br>**- T/D/Po/Pe (3 months)**: 95.0% (735/774)<br>**- T/D/Po/Pe (4 months)**: 94.6% (730/772)<br>**- MenC**: 91.7% (687/749)	Low

						**- Hib**: 92.9% (696/749)<br>**- Pneumococcal**: 89.2% (397/445)<br>**- MMR1**: 95.5% (715/749)<br>**- MMR2**: 89.3% (582/652)<br>**- T/D/Po/Pe (B1)**: 89.9% (586/652)<br>**- T/D/Po/Pe (B2)**: 69.2% (110/159)<br>**- MenC (teenage B)**: 58.8% (77/131)<br>**- HPV**: 81.8% (72/88)	
17.	Ellis et al. (2020)[17]	United Kingdom	Not specified	Qualitative (Focus group, Interviews) 2018	Gypsy, Roma and Traveller<br>- Adult women 15–54 years (n=7)	Mixed awareness of maternal vaccinations. <u>Not all participants had been offered vaccinations,</u> and there was hesitancy among those who were offered them.<br><br>*'If I wasn't pregnant I would have had it, but I don't know, I didn't like the thought of it when I was having the baby, no.'*<br>***Focus group interviewee***<br><br>Childhood immunisations were a means to keep their children healthy and participants ensured that <u>their children had all the infant vaccinations</u>.<br><br>*'The way I look at it, if you don't have it, if they did get something, it's your fault not getting this to save this baby. That's the way I look at it, one way or the other. It's like measles, everything can be dangerous, can't it?'*<br>***Individual interviewee 1***	High
18.	Elran et al. (2018)[18]	Israel	Not specified	Cross-sectional (Survey) 2014-2016	Religious groups<br>- Children 2 to 30 months (n=1,504 parents)	**Vaccination by Religiosity (n=1,347, p= 0.014)**<br>Orthodox (19.9%), Religious (32.9%), Traditional (33.2%), Secular (14.0%)<br><br>**Full simmunisation completion:** 97%<br>- Bedouins (99% of 393)<br>- Ultra-Orthodox Jews (98% of 378)<br>- Non-Bedouin Arabs (96 of 359)<br>- Other Jews (95% of 374)	Medium

19.	Ergönül et al. (2019)[19]	Turkey	BCG, DaPT/IPA/HiB, PCV, Hep A, HepB, MMR, Varicella, Tetatnus, diphtheria	Narrative Review 2012-2016	Refugees<br>- All ages, women and children 0 to 15 years (n=3,635,841)	**Vaccinations applied to Syrian children in camps and communities:** Year (number of vaccine given)<br>**- BCG (up to 3 months old):** 2014 (18,290), 2015 (34,356), 2016 (68,196)<br>**- DaPT/IPA/HiB:** 2014 (77,039), 2015 (131,098), 2016 (207,227), 2017 (451,594)<br>**- PCV:** 2014 (64, 922), 2015 (125,796), 2016 (203,524), 2017 (291,839)<br>**- HepA:** 2014 (11,847), 2015 (28,771), 2016 (33,949), 2017 (74,766)<br>**- HepB:** 2014 (59,743), 2015 (100, 244), 2016 (148,172), 2017 (269,085)<br>**- MMR :** 2014 (81,846), 2015 (105,069), 2016 (63,560), 2017 (192,268)<br>**- VAR (Varicella)**: 2014 (9,178), 2015 (22,728), 2016 (39,393), 2017 (860,395)<br>**- Td (Tetatnus and diphtheria):** 2014 (32,408), 2015 (34,704), 2016 (21,103), 2017 (27,430)<br><br>**Overall coverage estimate:** >95%	Low
20.	Fabiani et al. (2017)[20]	Italy	Rubella	Cohort (Retrospective) 2011-2015	Immigrants<br>- Adults (n=3,140 women)<br><br>Comparison:<br>- Italians (n=41,094 women)	**Vaccinated**<br>**- Migrants**: 25.2% (797/3,140)<br>**- Italians**: 40.2% (17,795/41,094)<br><br>**Rubella Immunization Rate (RIR)-ratio compared to Italians**<br>**- All immigrants:** (36.0% vs 60.2%, RIR-ratio= 0.60, CI: 0.57–0.63)<br>**- Recent immigrants** (RIR-ratio = 0.47, 95% CI: 0.42–0.53)<br>**- Immigrants from high migratory pressure countries (HMPC): Sub-Saharan Africa** (RIR- ratio = 0.41, 95% CI: 0.31–0.56), **Asia** (RIR-ratio = 0.42, 95% CI: 0.33–0.53)<br><br>**Unaware of rubella simmunisation status**: 56.8% immigrants, 35.3% Italians	Medium
21.	Fortunato et al. (2018)[21]	Italy	Influenza	Analytical cross-sectional 2009-2016	Disadvantaged areas (Deprivation)<br>- Adults ≥65 years (n= 152,770, as of 2015)	**Influenza vaccination coverage** averages 7 seasons: 2009-2010 to 2015-2016)<br>**By deprivation group:** Low (76.98%), Medium-low (76.97%), Medium (75.66%), Medium-high (68.72%), High (61.12%)	High
22.	Fougère et al. (2018)[22]	Switzerland	HBV	Cohort (Prospective, Serology) 2014-2017	Newly arrived migrants (Iraq, Syria, Eritrea)<br>- Children 1-5 years (n=200)	**Vaccination status (n=200)**<br>130 (65.0%) vaccination card before coming in Switzerland<br>100 (50.0%) Vaccination at birth<br>133 (66.5%) Vaccinated between 0 and 11 months<br><br>**Antibody response**<br>- 118 (59%) anti-HBs ≥ 1000 UI/L	High

						- 161 (81%) anti-HBs ≥ 100 IU/L (booster-type antibodies response)<br>- 39 (19%) anti-HBs < 100 IU/L<br>- 23 (11%) no detectable antibodies (<10 IU/L)	
23.	Fozouni et al. (2019)[23]	Germany	Measles<br>Polio<br>Tetanus	Cross-sectional (Survey)<br>2016	Refugee (Syria, Afghanistan, Iraq, Moldova<br>- Children aged 1-5 (n=219)	**Tempelhof** camp (n=179)<br>- 83 (47%) fully simmunised or partially simmunised and due to return<br>- 92 (51%) partially immunized<br>- 4 (2%) were unimmunised.<br><br>Neukölln camp (n=40)<br>- 2 (5%) were unimmunized<br>- 37 (92.5%) were partially immunised, none were partially simmunised and due to return<br>- 1 (2.5%) was fully immunised.	High
24.	Freidl et al. (2018) [24]	Netherlands	Diphtheria<br>Hepatitis A and B<br>Measles<br>MMR<br>Polio (type 1,2,3)<br>Tetanus<br>Varicella	Cross-sectional (Serology)<br>2016	Asylum seekers (Syria, Iran, Iraq, Afghanistan, Eritrea, Ethiopia)<br>- Adults (n= 622)	**Self-reported childhood vaccines:** Vaccinated (84.6%, 526/615), Not vaccinated (2.4%, 15/615), Did not know (11.9%, 74/615)<br><br>**Participant distribution:** Syria (n=297), Iran (n=109), Iraq (n=83), Afghanistan (n=75), Eritrea (n=56), Ethiopia (n=2)<br><br>**Prevalence of protective antibodies (seroprotection)**<br>**- Measles:** 88% (83-93%), all below 95% herd immunity threshold. Lowest Iran [overall (83.5%),18–25 years (70%)]<br>**- Mumps:** 91% (81-95%). All met 93% immunity threshold, except Iraq (81%); Insufficient (Syrian 18–25 years, Iraqi >25 years)<br>**- Rubella:** 94% (84-98%), Lowest Iraq [overall (84%),18–25 (88%)]; Insufficient [Syria 26–35 years (89.4%), Iraq 26–35 years (80%)]<br>**- Varicella:** 96% (92-98%), all above 91% threshold<br>**- Diphtheria:** 82% (65-88%). All met 80%, immunity threshold, except Afghanistan (65%); Full protection highest Iran (62%), lowest for Afghanistan (27%), Iraq (28%)<br>**- Tetanus:** 98% (86-100%), High for all countries (96% to 100.0%) except Eritrea [over all (86%), 36–45 years (79%). Full protection highest for Iran (96%), lowest for Eritrea (41%)<br>**- Polio type-1:** [91% (88-94%)]; Type-2 [95% (90-98%)], Type-3 [82% (76-86%)].<br>--- Only type-3 slightly below 86% herd immunity threshold Subgroups below threshold: Iraq 26–35-years (63% type-3), Eritrea 26–35-years (71% type-3, p= 0.027).<br>**- Hepatitis A:** 84% (54-100%), Lowest Iran [overall (53.7%), 18 to 35 years (42–44%)]	Low

						**- Hepatitis B (anti-HBs, vaccine induced immunity):** 27% (8-42%), Lowest Iraq (8%)	
25.	Fritschi et al. (2021)[25]	Switzerland	Tuberculosis	Cross-sectional (Prevalence) 2013-2019	Refugee (Eritrea, Somalia, Afghanistan, Brazil, Sudan)<br>- Children 0 to15 years (n=139)	**BCG vaccinated (written proof or scar) of notified cases:** Foreign born (57%, 17 cases), Swiss born (3%, 2 cases), Swiss Pediatric Surveillance Unit (SPSU) (20%, 19 cases)<br>**- Significant associated factors**: birth country (p=0.001), BCG vaccination status (<0.001)	Low
26.	Führer et al. (2016)[26]	Germany	Tetanus<br>Measles<br>Hepatitis B	Cross-sectional (Survey)<br>2015	Asylum seekers<br>- Adults and accompanying children (n=214)	**6.5 % (n = 14) of all respondents had a vaccination card**<br><br>**Adult vaccination status (n=214)**<br>**- Tetanus:** 28 % (n = 60)<br>**- Measles (at least two doses):** 21.1 % (n = 58)<br>**- Hepatitis B:** 15.4 % (n = 33)<br><br>**Accompanying children (n=unspecified)**<br>**- Tetanus: 40.7 % (n = 11)**<br>**- Measles (at least two doses): 33.3 % (n = 9)**<br>**- Hepatitis B: 22.2 % (n = 6)**	Low
27.	Ganczak et al. (2021)[27]	Poland	General	Qualitative (Focus groups, Interview)<br>2019	Migrants (Ukrainian)<br>- Adults 18 to 45 years (n=22)	54.5% (12/22) fully vaccinated in home country (Ukraine)<br>9% (2/22) fully vaccinated except for BCG<br>45.5% (10/22) report their children were fully vaccinated in Ukraine/Poland<br>9% (2/22) did not receive early vaccinations	Low
28.	Georgakopoulou et al. (2018)[28]	Greece	MCV<br>MMR	Cross-sectional (Surveillance)<br>2017-2018	Migrants (Roma)<br>- All ages (n=1,844)<br><br>Comparison<br>- Non-minority Greek (n=763)	**Roma cases:** 94.4% (1741/1844), unvaccinated, 5.6% one dose of MCV<br>**Non-minority Greek**: 72.6% (554/763) unvaccinated.	Low
29.	Glatman-Freedman et al. (2019)[29]	Israel	Influenza	Cross-sectional (Telephone survey)<br>2015-2016	Religious groups (Jews, Arabs)<br>- Children 1–18 years (n=1,040)	- **Arabs**: 37.7%(171/454)<br>- **Jewish**:23.0% (135/ 586)<br>- Vaccination highest among children 1–4 years of age in both the Jewish and the Arab population	Low
30.	Godefroy et al. (2018)[30]	France	Measles	Case series<br>2017	Migrants (Roma)<br>- All ages 6 months to 24 years (n=18)	18 cases of measles<br>83% (15/18) Not previously simmunised (unvaccinated)<br>17% (3/18)Vaccinated with 1st dose few days before onset of disease	Low
31.	Gorman et al. (2019)[31]	Scotland	Influenza, BCG	Qualitative<br>2018	Migrants (Polish)<br>- Adults late 20s–over60 years (n=13 females)	Vaccinated (children of females)<br>- 84.6% (11/13)<br>- All of those not vaccinated are single-children	Medium

32.	Gorman et al. (2020)[32]	Scotland	Influenza	Cross-sectional<br>(Survey)<br>2018	Migrants (Polish)<br>- Parents (n=128)	42% vaccinating<br>39% declining<br>90% long term UK resident	Low
33.	Habersaat et al (2020)[33]	Romania	Measles	Cross-sectional<br>(Telephone survey)<br>2018	Disadvantaged groups<br>- Persons 13 months–18years<br>- Ethnic minority (n=261)<br>- Ethnic majority (n=259)	**Vaccination status (n=520)**<br>- **Routine data:** 460 (88.5%) 0 dose, 47 (9.0%) 1 dose, 13 (2.5%) 2 doses<br>- **Self-reported:** 161 (31.0%) 0 dose, 354 (68.1%) 1 dose, 5 (1.0%) Do not know<br><br>Ethnic minorities disproportionally affected by measles. Most measles cases were unvaccinated and lived in low coverage areas.	Medium
34.	Hagstam et al. (2019)[34]	Sweden	Measles, Rubella	Cross-sectional<br>(Prevalence)<br>(Serology)<br>2014-2015	Immigrants (Asylum seekers)<br>- Adults (n=1,344)<br><br>Non-Asylum seekers (n=629)	**Asylum seekers**<br>**- Measles (n=969)**: Positive (85%, 827/969), Indeterminate (5%, 47/969), Negative (10%, 95/969)<br>**- Rubella (n=935):** Positive (94%, 884/935), Indeterminate (3%, 24/935), Negative (3%, 27/935)<br><br>**Antenatal care**<br>**- Measles (n=940):** Positive (70%, 654/940), Indeterminate (5%, 88/940), Negative (21%, 198/940)<br>**- Rubella (n=984):** Positive (87%, 858/984), Indeterminate (6%, 54/984), Negative (7%, 72/984)<br><br>**Measles (n=1,909)**<br>- Considerable differences by geographic origin (44–97%)<br>- **Highest measles seropositive:** Africa and Asia had highest median anti-measles IgG levels. Middle East and North Africa and Horn of Africa, 88% (783/886 and 172/195, respectively)<br>- **Lowest measles seropositive:** Baltic countries (44%, 17/39), the Newly Independent States and Russia (67%, 36/54), former Yugoslavia and Albania (68%, 115/168), remaining countries in Eastern Europe (72%, 86/119), and Latin America (75%, 27/36).<br><br>**Rubella (n=1,919)**<br>- Varied less between geographic regions (90–99%)<br>- **Highest rubella seropositive:** Eastern Europe (99%, 124/125),Western Europe (98%, 40/41), and the Baltic States (98%, 40/41). Middle East and North Africa (97%, 843/872) and from the Horn of Africa (95%, 188/198).<br>- **Lowest rubella seropositive:** Former Yugoslavia and Albania	Low

						(90%, 154/171), Asia (292/372, 91%), and sub-Saharan Africa (51/55, 93%).	
35.	Haider et al. (2019)[35]	Scotland	Measles, Mumps, Rubella, third dose of the primary vaccine (TPV), pre-school booster (PSB)	Cohort (Retrospective) 2008-2018	Disadvantaged areas (Low SES area)<br>- Children (n=329,897)	Uptake TPV and MMR 1 (>98.0%)<br><br>**- Ten-year average uptake:** Highest for the primary vaccine(99.2%), lowest for 2nd MMR dose (94.2%)<br>- Delay pronounced for 40% of most deprived population and immunisations scheduled at later ages.<br>- Uptake significantly associated with deprivation for all vaccines except the pre-school booster<br>- Differences in uptake for PSB when stratified by deprivation, especially for the middle class (deciles 4–7)	Low
36.	Hardelid et al. (2016)[36]	England Wales	Influenza	Cohort 2014 - 2015	Disadvantaged areas<br>- Children, 2 and 4 years (n=57,545)	38.7% (95% CI 38.3% to 39.1%) of 57 545 children were vaccinated against influenza<br><br>**Vaccination by deprivation group: (%, n/N)**<br>**- Most deprived:** 30.5% (2369/7757)<br>**- Least deprived:** 46.6% (6,113/13,110)<br><br>- 19% in poorest areas less likely to receive influenza vaccine compared to wealthiest (ARR=0.81, 95% CI (0.77 to 0.86)).	Medium
37.	Hudečková et al. (2020)[37]	Slovakia	Measles Mumps Rubella	Cross-sectional (Prevalence) 2018	Migrants (Roma)<br>- All ages 0–54 years (n=439)	>90% two-dose trivalent MMR vaccine<br>40.2% (175/435) unvaccinated or unknown vaccination status for measles<br><br>Measles vaccination status: 60.1% (264/439) vaccinated<br>- 31.2% (137/439) two doses<br>- 28.9% (127/439) one dose<br>- 35.3% (155/439) unvaccinated<br>- 4.6% (20/439) unknown<br><br>n=102 patients (with two-dose vaccination status) tested for antibodies against rubella and mumps<br>- **Measles**: 66.7% (68/102) positive IgM, 23 (22.5 %) IgG antibodies against measles.<br>- **Rubella**: 19.6% (20/102) seropositive IgG<br>- **Mumps**: 58.8% (60/102) seropositive IgG	Medium
38.	Hungerford et al. (2018)[38]	UK	Influenza	Analytical cross-sectional (Ecological) 2004-2016	Disadvantaged areas (Low SES)<br>- All ages (n=89,058)	Vaccine uptake lower than national targets in most neighbourhoods<br><br>**Odds of vaccine uptake in most deprived compared to least deprived:** 24–59 months [30% lower (OR 0.70; 95% CI 0.66 to	High

						0.74; p<0.001)], 65+ years [10% lower (OR 0.90; 95% CI 0.88 to 0.92; p<0.001)]	
39.	Hungerford et al. II (2018)[39]	UK	Rotavirus	Quasi-experiment (Interrupted Time-series Analyses)<br>2013-2016	Disadvantaged areas (Low SES)<br>- Children (n=18,259)<br><br>Comparison<br>- General population (n=31,836)	**Uptake of the 1st and 2nd (complete) dose of rotavirus vaccine**<br>**- Most deprived population:** 1st dose (90.6%, 16,550/18,259), 2nd dose (84.9%, 15,505/18,259)<br>**- General population:** 1st dose (91.4%, 29,108/31,836), 2nd dose (86.7%, 27,594/31,836)<br><br>**Relative risk between for most deprived compared to least deprived**<br>- **Risk of non-vaccination**: 1.54 (CI: 1.34–1.75)<br>- **Risk of non-completion 2-doses:** 1.97 (CI 1.62–2.41)	Low
40.	Hvass et al. (2019)[40]	Denmark	Polio	Cross-sectional (Prevalence)<br>(Serology)<br>2014 and 2016	Refugees<br>- All ages 6months-76 years (n=475)	96% (454/475) available vaccination data<br>94% Complete WPV immunity<br><br>-27 lacked antibodies against a least one serotype; 56.5% (17/23) followed complete simmunisation program (None lacked antibodies against all 3 polio types)<br>- 6% (10/168) children lacked antibodies and all were family-reunified refugees (no children from Danish asylum centre)<br>- Observed association between 20-30 years and lack of immunity against one or more polio types (p=0.001)<br>- Originating from The Horn of Africa, 20-30 years and male gender associated with lack of immunity	Medium
41.	Hvass et al. (2020)[41]	Denmark	Measles	Analytical cross-sectional<br>(Serology)<br>2016 - 2018	Refugees<br>- All ages (n=513)	84.8% (435/513) had immunity against measles<br>15.2% (78/513) lacked antibodies (evenly distributed between countries of origin)<br>Immunity increased with age, young children most vulnerable to infection(<19 years, 79.9%) vs (≥19 years, 89.1%)	High
42.	Iacoella et al. (2021)[42]	Italy	Tuberculosis, Hepatitis A and B, Influenza	Cross-sectional (Prevalence)<br>2021 (Based on population census with age distribution was from 2018)	Migrant (Homeless)<br>- Adults (n=112)<br>- Africa (n=28), Asia (n=6), South America (n=5), Europe (n=73, including Italy, n=34)	**Self-report immunization status (n=112):**<br>- **Tuberculosis:** Yes (18.8%), No (48.2%), Do not know (33%)<br>- **Hepatitis A**: Yes (15.2%), No (56.2%), Do not know (28.6%)<br>- **Hepatitis B:** Yes (17%), No (55.3%), Do not know (27.7%)<br>**- 2020-2021 Seasonal Influenza:** Yes (35.7%), No (64.3%), Do not know (0%)	Low
43.	Jablonka et al. (2017)[43]	Germany	Diphtheria<br>HBV<br>MMR<br>HCV<br>HIV<br>Syphilis	Cross-sectional (Serology)<br>2015	Refugees<br>- All ages 3–76 years (n=678)	**IgG levels for tetanus and diphtheria**<br><br>**Tetanus (n=678)**<br>- 43.7% sufficient IgG for long-term tetanus protection<br>- 56.3% insufficient IgG and no secure long-term protection<br>- 8.7% short-term protection from intermediate anti-tetanus IgG	Low

			Tetanus			but re-vaccination within next 2 years recommended<br>- 47.7% immediate tetanus simmunisation boost necessary due to low yet protective tetanus IgG levels (19.8%) or extremely low and non-protective anti-tetanus levels (27.9%).<br><br>**Diphtheria (n=678)**<br>- 23.9% sufficient IgG for long-term diphtheria protection<br>- 76.1% no long-term protection against diphtheria ((re-) vaccination needed)<br>- 2.1% complete seronegativity<br>- 45.6% low, unprotective diphtheria IgG<br>- 28.5% short-term protective IgG levels, immediate booster vaccine required	
44.	Jablonka et al. II (2017) [44]	Germany	MMV	Cross-sectional (Prevalence) (Serology) 2015	Refugees<br>- All ages (n=554)	**Overall seroprevalence % (CI); Complete protection (%)**<br>- **Measles (n=552):** 89.9% (CI: 87.3–92.4%); 88.5%<br>- **Rubella (n=554):** 86.6% (CI: 83.9–89.3%); 77.9%<br>- **Varicella (n=554):** 91.2% (CI: 88.8–93.3%); 95.9%<br><br>Disease specific seronegativity rates by top 5 countries (%, (95% CI))<br>**- Syria (n=243):** Varicella (1.6% (0.4–3.3), Rubella (13.2% (9.1–17.4), Measels (8.7% (5.2–12.3)<br>**- Afghanistan (n=76):** Varicella 9.2% (3.0–15.5), Rubella (5.3% (1.2–11.1), Measels (5.3% (1.2–11.1)<br>**- Iraq (n=42)**: Varicella (2.4% (0–8.5), Rubella (16.7% (6.1–28.6), Measels (16.7% (6.5–28.2)<br>**- Pakistan (n=39)**: Varicella (17.9% (6.8–31.2), Rubella (2.6% (0–8.6), Measels (2.6% (0–8.6)<br>**- Sudan (n=25):** Varicella (40% (21.1–60.0),Rubella (0), Measels 8% (0–20.0)<br><br>- 76.9% underage refugees and 68.4-75% migrants in the adult groups completely MRV-protected<br>- 27.9% of tested refugees showed incomplete seropositivity against all three diseases<br>- Females had slightly but insignificantly lower MRV protection rate (68.9% vs. 73% in males).<br>-Varicella immunity lowest in the youngest age group of both genders (seronegative): <18 years (10.1% male/4.5% female)	Low
45.	Jackson et al. (2017)[45]	United Kingdom	Diphtheria, tetanus, poliomyelitis, HPV, influenza, whooping cough	Cross-sectional Qualitative 2013-2015	Traveller communities<br>- All ages (n= 174)	Self-reported Immunisation status of participants (n=174)<br>- 33.9% (59/174) full vaccination<br>- 23.0% (40/174) partial vaccinated<br>- 6.3% (11/174) unvaccinated<br>- 36.8% (64/174) missing response	Low

						Self-reported Immunisation status of participants' children (n=174)<br>- 39.7% (69/174) full vaccination<br>- 9.8% (17/174) partial vaccinated<br>- 1.1% (2/174) unvaccinated<br>- 25.3% (44/174) not applicable<br>- 24.1% (42/174) missing response	
46.	Jenness et al. (2021)[46]	Norway	Measles	Cross-sectional (Prevalence)<br>2000-2016	Migrant (Somali)<br>- Children ≤ 2 years (n=11,334)	**Children born between 2000-2006 (n=11,334):** 87% (9855/1,334) vaccinated, 13% (1479/1,334) unvaccinated<br>**Measles vaccine coverage:** Children born to Somali parents (85%), National average (96%)<br>- **Annual vaccine coverage reduction:** boys (−0.7, [95%CI−0.8 to−0.5]), girls (−0.2 [95% CI−0.4 to−0.0]<br>- Mother's length of residency negatively associated with vaccine coverage ($p< 0.001$)<br>- Measles vaccine coverage associated with area of birth ($p< 0.001$)	Medium
47.	Jones et al. (2016)[47]	France	Measles	Cross-sectional (Surveillance)<br>2016	Refugees (Calais and Grande-Synthe settlements)<br>- Adult, Children (n=4,665)	**Measles vaccination**:<br>60% of target population (2,051/3,500) in Calais settlement<br>40% of eligible individuals (466/1,165) in the Grande-Synthe settlement	Medium
48.	Karasahin et al. (2021)[48]	Turkey	Hepatitis	Analytical cross-sectional<br>2011-2019	Migrants (Afghans)<br>- All ages (n=9,197)	**Hep-B virus infection and immune status (n=9,197):** HBV infection (7%, 505/9,197), Acquired immunity (2.8%, 201/9,917), Isolated anti-HBc IgG positivity (0.7%, 68/9,197), Natural immunity (1.8%, 127/9,197), Never exposed or vaccinated (87.5%, 6,295/9,197)<br><br>**Immune status by age**<br>**- Acquired immunity (n=201):** 0-15years (10.1%, 57/201), 16-25years (1.5%, 74/201), 26-40years (2.9%, 39/201), ≥41years (7.6%, 31/201)<br>**- Isolated anti-HBc IgG positivity (n=68):** 0-15years (0.7%, 4/68), 16-25years (0.9%, 46/68), 26-40years (0.8%, 15/68), ≥41years (0.7%, 3/68)<br>**- Natural immunity (n=127):** 0-15years (1.8%, 10/127), 16-25years (1.5%, 71/127), 26-40years (1.7%, 23/127), ≥41years (5.7%, 3/127)	Low
49.	Klok-Nentjes et al. (2018)[49]	Netherlands	Not specified	Cross-sectional (Survey)<br>2016	Undocumented (Brazilain, Other nationalities)<br>- Children (n=267)	88% undocumented children attending school vaccinated<br>50% of non-school-going children vaccinated<br><br>**Participants having undocumented minor children (n=39)**<br>- **Brazilian**: 95% (20/21) vaccinated<br>- **Other nationality**: 72% (13/18) vaccinated	Medium

50.	Köse et al. (2017)[50]	Turkey	Hepatitis A, B and C	Cross-sectional (Prevalence) (Serology) 2014-2015	Refugee (Syrian)<br>- Children 0-18 years (n=140)	**Seroprevalence sample for Hepatitis A, B, C and HIV**<br>- **HBsAg (n=140):** Positive (4.2%, 6/140), Negative (95.8%, 134/140),<br>- **Anti-HBs (n=140):** Positive (52.8%, 74/140), Negative (47.2%, 66/140)<br>- **Anti-HCV (n=109):** Positive (1.8%, 2/109), Negative (98.2%, 107/109)<br>- **Anti-HIV (n=88):** Positive (2.2%, 2/88), Negative (97.8%, 86/88)<br>- **Anti-HAV IgG (n=86):** Positive (47.7%, 41/86), Negative (52.3%, 45/86)<br><br>- HAV, HCV and HIV seroprevalence in Syrian refugee children similar to Turkish children; but HBsAg positivity more frequent and anti-HBs positivity less frequent	Low
51.	Letley et al. (2018)[51]	England	Childhood immunisations	Cross-sectional (Survey)<br>Qualitative (Interview) 2015-2016	Religious groups (Jews)<br>- Children (n=126)	**Survey (n=126)**<br>- 78 (62%) up-to-date<br>- 43 (34%) not up-to-date with immunisations<br>- 4 (3%) unsure status<br><br>**n=10 interviewed**<br>- 5 (50%) fully vaccinated.	Low
52.	Loiacono et al. (2020)[52]	UK	Influenza	Cohort (Retrospective) 2011-2016	Migrants (Ethnic minorities)<br>- Adults 18 to 65 years (n=3,391,975)	**Overall uptake**: 18–64 years (35.3%), 65+ years (74.0%)<br><br>**- Asian**: 18–64years (44.8%), 65+years (74.8%)<br>**- Black:** 18–64years (34.5%), 65+years (64.3%)<br>**- Mixed/Other:** 18–64years (36.3%), (65+years (76.2%)<br>**- Unknown:** 18–64years (31.8%), 65+years (70.6%)<br>**- White:** 18–64years (38.0%), 65+years (77.1%)<br><br>**Deprivation:**<br>- Low SES: decreased odds of being vaccinated among older adults (0.75 =R vs 0.93 OR for higher SES)	Medium
53.	Louka et al (2019)[53]	Greece Netherlands	**Adult** (Influenza, polio, tetanus, HBV)<br><br>**Children** (MMR, DTP, measles, mumps)	Cross-sectional (Survey)<br>Qualitative (Interview) 2017-2018	Asylum seekers, Refugees (Afghanistan, Syria, Eritrea)<br>- All ages (n=61)	**Adults (n=61):**<br>- 86.9% (53/61) vaccinated in country of origin.<br>- 19.7% (12/61) received additional vaccinations [influenza (n = 2), polio (n = 1), tetanus (n = 1), hepatitis B (n = 1), unknown (n = 7)]<br><br>**Participants' with children (n=34)**<br>- 91.2% (31/34) vaccinated in country of origin<br>- 79.4% (27/34) received additional vaccinations in the hosting countries (mostly MMR (n = 3), DTP (n = 3), measles (n = 2) and mumps (n = 1).	Low

						- 38.7% (12/31) could not recall which vaccines were given to the children.	
54.	Mazzitelli et al. (2021)[54]	Italy	Hepatitis B	Analytical cross-sectional (Serology) 2015-2018	Migrants (Bangladesh, Congo, Eritrea, Gambia, Guinea, Iraq, Ivory Coast, Libya, Mali, Nigeria, Pakistan, Senegal, Syria)<br>- All ages (n=330)	30% (99/330)received screening for HBV soon after their arrival in Italy (1st evaluation)<br><br>**Pre-intervention**<br>**- Screened group (n=99):** 6.1% HBSAg positive, 9.1% fully vaccinated, 64.6% eligible for vaccination, 11% natural immunization, 9.1% seronegative occult HBV infection OBI<br>- **Never screened (n=231) prescribed HBV testing after consultation:** 12.1% (28/231) refused further testing, 7.3% (17/231) were HBsAg postive, 13.6% (29/231) natural immunity, 7.3% (17/231) seronegative OBI, 59.2% (140/231) were seronegative.<br><br>**Intervention**<br>- After first consultation and HBV testing prescription, 204/330 (61.8%) migrants eligible for vaccination.<br>- 6 month follow-up: 56.9% (116/204) vaccinated, 17.6% (36/204) ongoing vaccination course, 17.1% (35/204) not started vaccination course, 8.3% (17/204) moved to other centres or were lost to follow-up<br>- 1 year of follow-up (n=142): 92% (131/142) completed HBV vaccination, 1.4% (2/142) vaccination course ongoing, 6.3% (9/142) not started the vaccination,<br>- At end (n=204): 30.4% (62/204) left their centres, 8.3% (17/204) still to complete the vaccination course.	Low
55.	Mellou et al. (2017)[55]	Greece	HAV	Cross-sectional (Prevalence) 2016	Refugees, asylum seekers, migrants (Syria, Afghanistan, Iraq)<br>- All ages (estimated population n=4,494)	1,681 refugees vaccinated at the five camps<br><br>64.4% (1,082/1,681) children mass vaccinated<br>8% (134/1,681) close contacts aged 1–14 years vaccinated during ring vaccination<br>12.1% (203/1,681) of close contacts aged 15 years or older vaccinated during ring vaccination	Low

56.	Mellou et al. (2019)[56]	Greece	MMR, DTaP, polio, pneumococcal, Hib, HBV	Cross-sectional (Prevalence) 2017-2018	Refugees, asylum seekers, newly arrived migrants (Afghanistan, Syrian, Iraqi)<br>- All ages	**57,615 vaccinated in 25 camps**<br>MMR (36.5%), DTP (12.74%), Polio (13.3%), Pneumococcal disease (10.3%), Hib (12.5%), Hepatitis B (12.5%)<br><br>**Vaccinated refugee children at 25 camps (n=3,786)**<br>- MMR (n=3,501): 1st dose (81.2%), 2nd dose (45%)<br>- DTP (n=1,509): 1st dose (46.3%), 2nd dose (24.5%)<br>- Polio (n=1,509): 1st dose (46.7%), 2nd dose (24.5%)<br>- Pneumococcal disease (n=1,509): 1st dose (49.7%), 2nd dose (17.7%)<br>- Hib (n=1,509): 1st dose (46.2%), 2nd dose (24.5%)<br>- Hepatitis B (n=1,509): 1st dose (48.6%), 2nd dose (24.7%)	Medium
57.	Mueller-Hermelink et al. (2018)[57]	Germany	BCG	Cross-sectional (Prevalence) 2015-2016	Asylum seekers (Syrian, Iraqi, Afghan, others)<br>- Children 0 to 15 years (n=1,379)	**BCG** vaccinated 57.6% (558/968)<br><br>**BCG Vaccinated children infected with TB**<br>**LTBI**: 58.6% (34/58)<br>**Active TB**: 62.5% (5/8)<br>**LTBI and active TB**: 39.1% (39/66))	Medium
58.	Mylius et al, (2019)[58]	Germany	Not specified	Cross-sectional (Retrospective database analysis) 2016–2018	Undocumented migrants<br>- All ages (n=236)	**People visiting a VS in 1st year of study (n= 236)**<br>- 3.8% (9 people) vaccinated 13 times (4 children, 4 women, 1 man)<br><br>**Advice seekers in 2nd year of study who received a treatment certificate (n=196):**<br>- 68% answered the questions about vaccinations<br>- 59% could not remember having a vaccination document<br>- 18% remembered a vaccination document but unsure where it was<br>- 23% had it according to their own information, sure about one	Low
59.	Nakken et al. (2018)[59]	Denmark	DTaP/IPV/Hib, Prevnar/Pneumococcal<br>MMR	Cross-sectional (Retrospective database analysis) 2015	Asylum seekers (Syrian, Afghan, Stateless Palestinians, Iranian, Iraqi, Eritrean Somali)<br>- Children, Adolescents (n=2,126)	1328 vaccinations after health screening<br>- 60% adequately vaccinated<br>- 7% partly vaccinated<br>- 33% inadequate vaccinations compared to national guidelines<br><br>**Unvaccinated or unknown vaccination:**<br>- Boys (37%, 499/1350) vs Girls (27%, 209/776)<br>- 0- to 5-year olds (22%, 122/556), 6- to 11- year olds (26%, 189/728), 12 and 17 years (48%, 404/842)<br><br>**Received lowest number of total vaccinations distributed**: Iraqis (5%), stateless Palestinians (7%), Iranians (7%) and Eritreans (7%)	High

						**Least likely to be vaccinated**: Afghans (57%) and Eritrean (54%)	
60.	Natan et al. (2016)[60]	Israel	Influenza	Cross-sectional (Survey) 2015	Muslims<br>- Parents of children ≤12 years (n=200)	**Parents who vaccinated their children** were younger (M = 32, SD = 5.7) than parents who did not vaccinate their children (M = 32, SD = 5.7) (t(4.0), df = 198, p < 0.05).<br><br>**Parents who vaccinated their children** had fewer children (M = 2.3, SD = 0.9) than parents who did not vaccinate their children (M = 2.7, SD = 1.0) (t(3.2), df = 198, p < 0.05).	Low
61.	Nidzvetska et al. (2017)[61]	Ukraine	Not specified	Qualitative (Semi-structured interviews) 2016	Internally displaced persons<br>- Adults (n=9 mothers)	**General lack of routine child vaccinations after displacement**<br><br>- Women taking their children for postnatal check-ups and simmunisation found no vaccines in stock.<br>- Reason associated with a lack/rupture of stocks was poor economic state of the country following the conflict.<br>- Waiting for vaccines takes from one month to more than a year<br><br>*"My child has not received a single vaccine, since they were not available. We missed an opportunity to get vaccination when it was there for one week, and now they told us to wait for another year" (resp. 4)*	Medium
62.	Norman et al. (2021)[62]	Spain	MMR, Varicella (VZV), Hepatitis A and B	Cross-sectional (Prevalence) (Serology) 2018-2019	Migrants<br>- Adults (n=468)	468 patients: Africa (52.5%, of which 88.2% from sub-Saharan Africa), Latin America (38.5%), Other areas (9%)<br><br>**Positive seroprevalence**<br>**- Rubella:** Overall (90.5%, 380/420); Africa (91.6%, 219/239), Latin America (87.4%, 125/143)<br>**- Measles:** Overall (83.3%, 339/407); African (93.3%, 224/240), Latin America (82.1%, 115/140)<br>**- Mumps:** Overall (83.3%, 339/407); Africa (90.1%, 9209/232), Latin America (75.9%, 104/13)<br>**- Varicella:** Overall (91.4%, 308/337); >90% in migrants from all areas.<br>**- Hepatitis B:** immunised (16.9%, 77/456), positive HBV surface antigen (5.9%, 27/456), evidence of past infection (28.3%, 129/456)<br>**- Hepatitis A:** Overall (89.7%, 402/448); Africa (97.1%, 234/241), Latin America (82.7%, 139/168)	Low

63.	Öztaş et al. (2020)[63]	Turkey	BCG,<br>CPV<br>HAV<br>HBV<br>MMR<br>OPV<br>Varicella<br>**Five-component combined vaccine**	Cross-sectional (Survey)<br>2017	Migrants (Syria)<br>- Children (n=2,827)	2,193 (77.6%) at least one vaccination<br>634 (22.4%) never vaccinated<br><br>**Appropriate immunization doses:**<br>- **BCG**: 36.1% (n = 1,020)<br>- **CPV**: 24% (n = 685)<br>- **HAV**: 5.4% (n = 154)<br>- **HBV**: 26.5% (n = 750)<br>- **MMR**: 3.8% (n = 107)<br>- **OPV**: 11.3% (n = 320)<br>- **Varicella**: 21.1% (n = 596)<br>- **Five-component combined vaccine:** 25.5% (n = 722)<br><br>**Never vaccinated immunizations**:<br>- **BCG**: 62.5% (n = 1,768);<br>- **CPV**: 58% (n = 1,641)<br>- **HAV**: 76.0% (n = 2,148)<br>- **HBV**: 54.7% (n = 1,547)<br>- **MMR**: 76.6% (n = 2,166)<br>- **OPV**: 70.8% (n = 2,001)<br>- **Varicella**: 66.8% (n = 1,889)<br>- **Five-component combined vaccine:** 64.6% (n = 1,826)	Low
64.	Perniciaro et al. (2018)[64]	Germany	Invasive pneumococcal disease (IPD)	Case-control (Retrospective, Unmatched)<br>2014-2017	Refugee<br>- Children (n=21)<br><br>Comparison<br>- German born children (n=405)	(PCV13 vaccinated vs unvaccinated)<br><br>**Refugee children (n=21): (9%, 2/21) vs (86%, 18/21)**<br>**By year:**<br>- **2014–15 (n= 3):** All unvaccinated<br>- **2015–16 (n= 12):** (17%, 2/12) vs (83%, 10/12)<br>- **2016–17 ((n= 6):** All unvaccinated<br><br>**Germany-born children (n=405): (68%, 276/405) vs (21%, 85/405)**<br>**By year:**<br>- **2014–15 (n= 107):** (68%, 73/107) vs (18%, 19/107)<br>- **2015–16 (n= 122):** (70%,86/122) vs (17%, 21/122)<br>- **2016–17 ((n= 176):** (66%, 117/176) vs (26%, 85/176)<br><br>*- Study also reports VT (Vaccine type) serotype, Non-VT serotype, Resistant to >3 classes of antimicrobial drugs (data not extracted)*	Low
65.	Perry et al. (2020)[65]	Wales	MMR, DTP, meningococcal	Cross-sectional (Prevalence)<br>2014-2018	Asylum seekers<br>- Children (n=388)<br><br>Comparison: Local children (n=56,473)	**Asylum-seeking children (n=388):** Measles 73.7% (286/388), Tetanus 73.5% (285/388), Men C 76.0% (295/285)<br>**Local children (n=56,473):** Measles (89.4%), Tetanus (89.3%), Men C (88.3%)<br><br>- **Significant difference (P< 0.05) in coverage:** Measles (15.6%), tetanus (15.9%), Men C (12.3%)	Low

66.	Perry et al. II (2020)[66]	Wales	Pertussis	Analytical cross-sectional<br>2013-2017	Disadvantaged areas (Low SES)<br>- Children (n=163,733)	**Overall uptake:** 1st dose by 12 weeks (87.9%), 3 doses by 24 weeks (87.1%), 3 doses by 52 weeks (96.3%)<br>**- 3 doses uptake by deprivation:** Most deprived (97.1%), least deprived (98.2%)<br>--- **Uptake decrease:** Most deprived (Year 2013, 97.8%) to ((Year 2017, 95.5%), Least deprived (Year 2013, 98.3%) to (Year 2017, 97.6%)<br><br>**Vaccination timeliness by deprivation:**<br>- **3 doses on time:** Most deprived: [73.2%(2013) – 76.9% (2017)] vs least deprived (83.1% – 87.3%)<br>- **3 doses by 20 weeks:** Most deprived (63.8%) vs least deprived (79.6%)<br>- **3 doses by 24 weeks:** Most deprived (81.5%) vs least deprived (91.9%)	High
67.	Pohl et al. (2017)[67]	Switzerland	General	Cross-sectional (Prevalence)<br>2015	Refugee<br>- Children (n=93)	93 of 105 admissions analysed<br>58.1% (54/93) sImmunisation status not documented in medical records<br>35.5% (33/93) Vaccine status was unknown<br>6.5% (6/93) Vaccination status documented<br>3.2% (3/99) Received vaccination during hospitalisation	Low
68.	Rath et al (2018)[68]	Germany	Not specified	Cross-sectional (Survey)<br>2015-2016	Migrants, Asylum-seekers<br>- Children (n=405)	73% with up-to date on immunisations<br>39% Received scheduled simmunisations since migration<br>22% with vaccination record<br>46.4% lost vaccination record during migration	Medium
69.	Sane et al. (2016)[69]	Finland	Diphteria	Case report<br>2015	Asylum seeker<br>- All ages (n=30)	30 exposed residents<br>66.7% (20/30) vaccinated against diphtheria, mostly adolescents from Afghanistan	Medium
70.	Serre-Delcor et al. (2018)[70]	Spain	HBV	Cross-sectional (Retrospective)<br>2013-2016	Asylum seekers<br>- All ages (n= 303)	82.5% (250/303) preliminary test conducted<br>66.2% (192/290) HBV vaccination indicated<br><br>**Dose among those that reported being vaccinated** (n=192)<br>3-doses (21.9%), 2-doses (34.4%), 1-dose (21.9%), Did not start vaccination schedule (21.9%)	Medium
71.	Shahbabi et al. (2021)[71]	Israel	Influenza, HPV	Cross-sectional (Prevalence)<br>2019	Religious and ethnic groups (Northern Bedouin, Druse , Muslim, Arab - Christians , secular Jewish , traditional Jewish)<br>- Adults, mothers (n=693)	**Uptake by religion**<br>**- Influenza vaccine:** Bedouin (74%), Druze (74%), Muslim (60%), Religious Jews (26%), Secular Jews (38%)<br>**- HPV:** Bedouin (99%), Druze (92%), Muslim (92%), Religious Jews (33%), Secular Jews (53%)<br><br>**Uptake by ethnicity**	Medium

						**- Arabs:** Influenza (62%), HPV (90%)<br>**- Jewish:** Influenza (34%), HPV (46%),	
72.	Staehelin et al. (2019)[72]	Switzerland	TD, MMR, VZV, Hep B	Cross-sectional (Prevalence) (Serology) 2016-2017	Asylum seekers (Eritrean)<br>- All ages (n= 133)	133 study participants (20 women, 113 men)<br>**Hepatitis B status:** Immunity from vaccination (0.8%), Susceptibility (69.9%), Existing chronic hepatitis B (1.5%), Immunity from previous infection (21.8%)<br><br>**Seropositivity by gender (gender difference non-significant)**<br>**- Diphtheria:** women (57.9%), men (74.8%) (non-significant)<br>**- Tetanus:** women (94.8%), men (41.1%) (significant P<0.001)<br>**- Measles:** women (73.7%), men (76.6%) (non-significant)<br>**-Varicella:** women (89.5%), men (95.3%) (non-significant)<br>**- Anti-HBc:** women (15.8%), men (26.2%) (non-significant)<br>**- Anti-HBs:** women (15.8%), men (17.8%) (non-significant)<br>**- Rubella:** only women (78.9%)	Low
73.	Stein-Zamir et al. (2017)[73]	Israel	MMR,MMRV, DTaP	Qualitative (Focus groups, Interviews) 2015	Religious group (Jewish ultra-orthodox)<br>- Adults (n= 87 Mothers)<br><br>Comparison<br>- District coverage rate	**Jewish ultra-orthodox**<br>**- DTaP4 vaccine** (77–82%)<br>- **MMR1\ MMRV1 vaccine** (91–94%)<br><br>**District coverage**<br>- **Low DTaP4 vaccine** (89%)<br>- **Low MMR1\ MMRV1** (96%)	Medium
74.	Stein-Zamir et al. (2019)[74]	Israel	HBV3, DTaP-IPV-Hib4, PCV3, MMR/MMRV1, HAV1 and HAV2	Cohort (Prospective) 2009-2016	Religious groups (Jews)<br>- Children ≤ 7 years (n=3,098)	**Age-specific vaccination rates different points:**<br><br>- **HBV3**: 31.5%, 82.8%, 90.8% (at 7, 12, 24 months)<br>- **DTaP-IPV-Hib4**: 27.7%, 64.8%, 80.2% (at 13, 18 and 24 months)<br>- **PCV3**: 37.6%, 64.1%, 72.6% (at 13, 18, 24 months)<br>- **MMR/MMRV1**: 58.3%, 85.2%, 90.8% (at 13, 18, 24 months)<br>- **HAV1**: 48.6%, 78% (at 19, 24 months)<br><br>**48 months:** 82–95% children up-to-date<br>**HBV3** (94%), **DTaP-IPV-Hib4** (91%), **PCV3** (79%), **MMR/MMRV1** (95%), **HAV1** (92%), **HAV2** (82%)<br><br>**7 years:**<br>**HBV3** (1.7%), **DTaP- IPV4** (3.1%), **PCV3** (1%), **MMR/MMRV1** (1.2%), **HAV2** (8%)	High

75.	Suppli et al. (2018)[75]	Denmark	MMR, HPV	Cohort<br>(Register-based)<br>2014-2015	Migrant<br>- Children (n=9,692 girls 14 years)	**Uptake of Vaccination by mothers' ethnicity (n=9692):** Non-western immigrant (11.9%), Western immigrant (2.4%), Danish born (85.7%)<br><br>**Odds for vaccination for compared to Danish born (OR (95% CI)**<br>- **Lacking only MMR (n=2026, 173 MMR vaccine administered):** Non-western [1.01 (CI: 0.59–1.74)], Western (not reported)<br><br>**- Lacking both HPV and MMR (n=2726)**<br>**--- 121 MMR vaccine administered:** Non-western [1.0 (0.47–2.11)], Western [1.65 (0.58–4.72)]<br>**--- 148 MMR & HPV vaccine administered**: Non-western (1.54 (CI: 0.87–2.72), Western [1.54 (0.59–3.99)]	High
76.	Tayfur et al. (2019)[76]	Turkey	Measles, Polio	Cross-sectional<br>(Prevalence)<br>(Retrospective Observational Registry)<br>2011-2016	Refugees (Syrian)<br>- All ages (n=2,854,968)	**- Living in the camps unvaccinated:** Polio (25%), Measles (33%)<br>**- Living outside the camps unvaccinated:** Polio (45%), Measles (41%)	Medium
77.	Taylor et al. (2019)[77]	England	HBV	Cross-sectional<br>(Survey)<br>2013-2015	Hard-to-reach populations<br>- All ages (n=346)	52.3% (**/346) full vaccine course (3 doses)<br>65.6% (227/346) at least one dose<br><br>**From vaccinated (n=227)**<br>**3-doses** (79.7%), 2-**doses** (11.5%), **1-dose** (6.6%)<br><br>**HBV vaccination (n=346):** Complete (52.3%, 181/346), Incomplete (37.6%, 130/346), Unknown (10%, 35/346)<br>**By ethnicity**:<br>**- Black African (n=25)**: Complete (36%, 9/25), Incomplete (64%, 16/25)<br>**- Black Other (n=45):** Complete (60%, 27/45), Incomplete (35.6%, 16/45), Unknown (4.4%, 2/45)<br>**- White (C/E-Central and Eastern)European (n=82):** Complete (45.1%, 37/82), Incomplete (40.2%, 33/82), Unknown (14.6%, 12/82)<br>- **White Other (n=160):** Complete (55.6, 89/160), Incomplete (33.1%, 53/160), Unknown (11.3%, 18/160)<br>- **Mixed/Other (n=32):** Complete (56.3%, 18/32), Incomplete (34.4%, 11/32), Unknown (9.4%, 3/32)<br>-**Unknown (n=2):** Complete (50%, 1/2), Incomplete(50%, 1/2)<br><br>--- Being female associated with lower vaccine uptake (2.37 [1.24–4.57], 0.01)	High

						--- Intravenous drug use associated with protection against incomplete HBV vaccination (p = 0.004)<br>--- Common reasons for incomplete vaccination: never being offered vaccine or not returning for further doses	
78.	Tchidjou et al. (2018)[78]	Italy	Not specified	Cross-sectional (Serology) 2012-2016	Adopted<br>- Children (n=108)	55.6% (60/108) at least one dose of vaccine<br><br>51.1% (24/47) of non-vaccinated children were more than 6 years (p=0.149)	High
79.	Tessier et al. (2018)[79]	UK	Influenza	Cross-sectional (Prevalence) 2015-2017	Disadvantaged areas, Religious groups<br>- All age (n=7,596)	**General Vaccine uptake:**<br>- **Children 2-4years**: 2015/16 (31.5%), 2016/17 (34.5%)<br>**- 16 to <65years:** 2015/16 (45.5%),2016/17 (49.4%)<br>**- ≥65year:** 2015/16 (71.0%), 2016/17 (70.6%)<br>**- All pregnant women:** 2015/16 (42.3%),2016/17 (44.9%)<br><br>**Crude uptake (%)**<br>**- By deprivation:**<br>**--- Most deprived:** 2015/16 (26.7%), 2016/17 (29.0%)<br>**--- Least deprived:** 2015/16 (39.5%), 2016/17 (42.9%)<br><br>**- By Ethnicity**<br>**--- Black minority ethnicity quartiles (34+% vs <5% LSAO):** 2015/16 (25.4% vs 36.6%), 2016/17 (27.8% vs 39.1%)<br><br>**By religion**<br>**- Jewish (>0% vs 0% LSOA):** 2015/16 (29.5% vs 32.0%), 2016/17 (32.7% vs 35.0%)<br>**- Muslims (6+ vs 0% LSOA):** 2015/16 (26.1% vs 36.5%), 2016/17 (28.6% vs 39.6%)	High
80.	Van Den Heuvel R. et al. (2018)[80]	Germany	Measles	Cross-sectional (Prevalence) 2018	Asylum Seekers (Turkey, Syria, Iraq, Iran, Nigeria, Afghanistan, Guinea, Albania, Somalia, Pakistan)<br>- Ages 1-49 year (n=4,606)	73% Measles immunization rate<br><br>**- sImmunisation rate by age cohorts:** 1-4 years of age (67%), 10-14 years (78%)<br>**- Main reasons for non-vaccination against measles:** refusal (21%), being ill (2%), pregnancy (2%), "other" (1%)	Low

81.	Veronesi et al. (2019)[81]	Italy	Polio	Cross-sectional (Prevalence) (Serology) 2004-2017	Migrants<br>- All ages (n=2,138)	2,138 blood samples collected<br><br>**Seroprotection**<br>**Antibody titers below ≥1:8:** poliovirus type-1 (94.0%), type-2 (88.4%), type-3 (15.0%)<br>**Antibody titers below <1:8:** poliovirus type-1 (6.0%), type-2 (7.7%), type-3 (15%)<br>**All strains:** Triple positive (79.2%), Triple negatives (1.3%)).<br><br>**Protection against 3 types :** 79.2%<br>**WHO region :** AMR (75.4%), AFR (79.6%), SEAR (87,4%), EUR (70%), EMR (81.5%), WP (80.1%)<br>- Non-seroprotection stratifying by WHO region of origin: WPR highest against poliovirus 1 (8.8%), European Region highest against polio 2 and 3 (respectively 11.7% and 24.6%).	Low
82.	Vita et al. (2019)[82]	Italy	Hexavalent, MMR, Pneumococcal, MCV, HBV, diphteria, tetanus, pertussis, polio, MMRV	Cross-sectional (Database review) 2013-2017	Asylum seekers (Africans, Asians)<br>- All ages (n=3,941)	85% (3,350/3,941) vaccinated<br>4252 vaccinations administered (95% of minors, 85% of adults)<br><br>**Children vaccinated (n=112)**<br>- **Hexavalent vaccine** (84%), **MMRV** (100%), **Pneumococcal** (94%), **MCV** (86%), **HBV** (53%), **DTaP-IPV** (31%), **Polio** (6%)<br><br>**Adults vaccinated (85%, n=3249)**<br>- **Polio** (94%), **Varicella zoster** (4.5%), **Pneumococcal** (3.1%), **DTaP-IPV** (1%)<br><br>**When vaccines were delivered directly upon arrival in the centre**<br>- 10.5% vaccinated in the first three years (2013 -2015)<br>- 66% in the last year (2016-2017)	Low
83.	Vu et al. (2020)[83]	Switzerland	Hepatitis A and B	Cross-sectional (Prevalence) (Serology) 2015- 2016	Immigrants (Sub-Saharan Africa, Eastern Europe)<br>- Adults ≥18 years (n=96)	96 female sex workers: predominantly undocumented immigrants (60%) from Africa and Eastern Europe with no health insurance, one participant (1%) Swiss-born.<br><br>**History of hepatitis B vaccination:** Yes (20%, 19/96), No (63%, 61/96), Does not know (17%, 16/96)<br>**Received combined hep-A and hep-B vaccine:** Yes (76% , 73/96), Not needed (24%, 23/96)	Low
84.	Ward et al. (2017)[84]	UK	Shingles/VZV	Cohort 2014/2015	Migrants (Ethnic minorities)<br>- Adults ≥70 years, general population (n=502,058)	35.6% (178,808/502,058) eligible adults had ethnicity recorded<br>**Crude vaccine coverage:** 59.5% (95%CI: 59.3–59.7<br>**Coverage by deprivation:** most deprived (54,1%) vs least deprived (64.1%)<br>**Coverage by ethnicity:** Black Caribbean (42.1%), Black-African	Medium

						(43.1%), Black other (40.2%), Chinese (45.2%), Indian (5.7%), Pakistani (49.2%) White-British (60.7%, CI: 60.5–61.0)	
85.	Watkinson et al. (2022)[85]	UK	Influenza	Cohort (Retrospective)<br>1 December 2020 - 18 April 2021	Migrants (Ethnic minorities)<br>- Adults ≥18 years (n=1,099,503)	**Influenza vaccine uptake:** 55.71% (419,314/752,715) eligible<br><br>**Ethnic minorities (compared to White British group)**<br>- Inequalities in vaccine uptake were widest amongst 'White and Black Caribbean'(HR 0.63, 95% CI 0.58 to 0.68) and 'White and Black African'(HR 0.67, 95% CI 0.63 to 0.72).<br>- Uptake slightly higher in other ethnic group (HR1.11,95% CI 1.09 to 1.12) and Bangladeshi (HR1.08,95%CI1.05to 1.11).<br>Overall, ethnic inequalities in vaccine uptake were wider for COVID-19 than influenza vaccination for 15 of 16 minority ethnic groups.<br>COVID-19 vaccine uptake inequalities also existed amongst individuals who previously took up influenza vaccination.	Medium
86.	Werber et al. (2017)[86]	Germany	MMR	Cross-sectional (Surveillance)<br>2014-2015	Asylum seekers (Bosnia and Herzegovina, Serbia, Syria, Others)<br>- All ages (n=32 homes)	1,344 cases of measles<br>Available vaccine information (n = 1,258)<br>86% (n=1,086) unvaccinated<br>12% (146/1,258) asylum seekers unvaccinated<br><br>**Post-exposure vaccination evaluation in n=32 asylum seekers homes**<br>- 7/32 no detailed information<br>- 7/32 no post-exposure intervention performed<br>- 3/18 home vaccination within recommended 72h after detection of measles<br>- 15/18 homes, 16 cases notified<br>- 47% (1,133/2,390) vaccination offered to all inhabitants in 8 homes<br>- 53% (706/1,344) reached	Medium
87.	Yakut et al (2020)[87]	Turkey	Influenza<br>Pertussis	Cross-sectional (Survey)<br>2015-2016	Disadvantage (**Low socioeconomic group)**<br>- Adult pregnant women (n=465)	**Acceptance rates (n=465)**<br>- **Influenza** (19.8%), **Pertussis** (11.2%)	Medium

NOTE:

B1 = booster 1
B2 = booster 2
BCG = Bacillus Calmette–Guérin vaccine
CPV = Conjugated Pneumococcal Vaccine
DTaP = diphtheria, Tetanus, acellular Pertussis
DTP = Diphtheria, Tetanus, Pertussis
GRT = Gypsy, Roma, and Traveller communities
HAV = Hepatitis A vaccine
HBcAB= total Hepatitis B core antibody
HbeAb = Hepatitis B e antibodies
HbeAg = Hepatitis B e antigen
HBsAb = Hepatitis B antibody
TST = Tuberculin Skin Test
HbsAb = Hepatitis B surface antibodies
HBsAg; Hepatitis B surface antigen
HBV = Hepatitis B vaccine
HCV = Hepatitis C vaccine
HCVAb = Hepatitis C virus antibodies
Hexavalent vaccine = Diphtheria, Tetanus, Pertussis, Poliomyelitis, Hemophilus Influenza, Hepatitis B)
Hib = Haemophilus influenzae type B vaccine
HIV = Human Immunodeficiency Virus
HPV = Human Papillomavirus
IgG = Immunoglobulin G antibody
IPV = Inactivated polio vaccine
LTBI = Latent TB Infection
MCV = Meningococcal vaccine
MenC = Meningitis C vaccine
MMR = Measles, Mumps, Rubella Vaccine
MMR1 = First dose of measles-mumps-rubella vaccine
MMR2 = Second dose of measles-mumps-rubella vaccine
MMRV = Measles-Mumps-Rubella-Varicella Vaccine
NIP = National Immunisation Program
PCV = Pneumococcal Conjugate Vaccine
OR = Odds Ratio
T/D/Po/Pe = Tetanus, Diphtheria, Polio, Pertussis
TB = Tuberculosis
Teenage B = Teenage Booster
VCR = Vaccination Coverage Rate
PSB = Preschool booster
AFR = African Region
AMR = Region of the Americas
SEAR =South-East Asia Region
EUR = European Region
EMR = Eastern Mediterranean Region
WHO = World Health Organisation
WPR = Western Pacific Region
LSOA = Lower Layer Super Output Areas

References List – Included Non-COVID studies

1. Affanni P, Colucci ME, Capobianco E, et al. Immunity status against tetanus in young migrants: a seroprevalence study. *Acta Biomed*. 2020;91(3-S):77-84. doi:10.23750/abm.v91i3-S.9438
2. Ahmad BB, Kristensen KL, Glenthoej JP, et al. Latent tuberculosis infection among minor asylum seekers in Denmark. *Eur Respir J*. 2020;55(1):1901688 doi:10.1183/13993003.01688-2019
3. Bechini A, Pieralli F, Chellini E, et al. Application of socio-economic-health deprivation index, analysis of mortality and influenza vaccination coverage in the elderly population of Tuscany. *J Prev Med Hyg*. 2019;59(4 Suppl 2):E18-E25. doi:10.15167/2421-4248/jpmh2018.59.4s2.1116
4. Bell S, Saliba V, Ramsay M, Mounier-Jack S. What have we learnt from measles outbreaks in 3 English cities? A qualitative exploration of factors influencing vaccination uptake in Romanian and Roma Romanian communities. *BMC Public Health*. 2020;20(1):381.. doi:10.1186/s12889-020-8454-x
5. Bielecki K, Kirolos A, Willocks LJ, Pollock KG, Gorman DR. Low uptake of nasal influenza vaccine in Polish and other ethnic minority children in Edinburgh, Scotland. *Vaccine*. 2019;37(5):693-697. doi:10.1016/j.vaccine.2018.11.029
6. Bielecki K, Craig J, Willocks LJ, Pollock KG, Gorman DR. Impact of an influenza information pamphlet on vaccination uptake among Polish pupils in Edinburgh, Scotland and the role of social media in parental decision making. *BMC Public Health*. 2020;20(1):1381.. doi:10.1186/s12889-020-09481-z
7. Boddington NL, Warburton F, Zhao H, et al. Influenza vaccine effectiveness against hospitalisation due to laboratory-confirmed influenza in children in England in the 2015-2016 influenza season - a test-negative case-control study. *Epidemiol Infect*. 2019;147:e201. doi:10.1017/S0950268819000876
8. Boukamel M, Fougère Y, Gehri M, et al. Prevalence of tuberculosis in migrant children in Switzerland and relevance of current screening guidelines. *Swiss Med Wkly*. 2020;150:w20253.. doi:10.4414/smw.2020.20253
9. Brockmann SO, Wjst S, Zelmer U, et al. ÖGD-Initiative zur Verbesserung der Durchimpfung bei Asylsuchenden [Public Health initiative for improved vaccination for asylum seekers]. *Bundesgesundheitsblatt Gesundheitsforschung Gesundheitsschutz*. 2016;59(5):592-598. doi:10.1007/s00103-016-2335-6
10. Burström B, Mellblom J, Marttila A, et al. Healthcare utilisation and measles, mumps and rubella vaccination rates among children with an extended postnatal home visiting programme in a disadvantaged area in Stockholm, Sweden-A 3-year follow-up. *Acta Paediatr*. 2020;109(9):1847-1853. doi:10.1111/apa.15176
11. Byrne L, Ward C, White JM, Amirthalingam G, Edelstein M. Predictors of coverage of the national maternal pertussis and infant rotavirus vaccination programmes in England. *Epidemiol Infect*. 2018;146(2):197-206. doi:10.1017/S0950268817002497
12. Ceccarelli G, Vita S, Riva E, et al. Susceptibility to measles in migrant population: implication for policy makers. *J Travel Med*. 2018;25(1):10.1093/jtm/tax080. doi:10.1093/jtm/tax080

13. Cuomo G, Franconi I, Riva N, et al. Migration and health: A retrospective study about the prevalence of HBV, HIV, HCV, tuberculosis and syphilis infections amongst newly arrived migrants screened at the Infectious Diseases Unit of Modena, Italy. *J Infect Public Health*. 2019;12(2):200-204. doi:10.1016/j.jiph.2018.10.004
14. Dam Larsen F, Jespersen S, Wejse C, Petersen E, Larsen CS. One-sixth of inpatients in a Danish infectious disease ward have imported diseases: A cross-sectional analysis. *Travel Med Infect Dis*. 2017;20:43-48. doi:10.1016/j.tmaid.2017.10.012
15. Decuyper II, Van Damme P, Soentjens P, Wojciechowski M. Low adherence with national travel medicine recommendations in Belgian expatriate children: A retrospective analysis. *Travel Med Infect Dis*. 2019;101424. doi:10.1016/j.tmaid.2019.05.010
16. Dixon KC, Mullis R, Blumenfeld T. Vaccine uptake in the Irish Travelling community: an audit of general practice records. *J Public Health (Oxf)*. 2017;39(4):e235-e241. doi:10.1093/pubmed/fdw088
17. Ellis N, Walker-Todd E, Heffernan C. Influences on childhood immunisation decision-making in London's Gypsy and Traveller communities. *Br J Nurs*. 2020;29(14):822-826. doi:10.12968/bjon.2020.29.14.822
18. Elran B, Yaari S, Glazer Y, Honovich M, Grotto I, Anis E. Parents' perceptions of childhood simmunisation in Israel: Information and concerns. *Vaccine*. 2018;36(52):8062-8068. doi:10.1016/j.vaccine.2018.10.078
19. Ergönül Ö, Tülek N, Kayı I, Irmak H, Erdem O, Dara M. Profiling infectious diseases in Turkey after the influx of 3.5 million Syrian refugees. *Clin Microbiol Infect*. 2020;26(3):307-312. doi:10.1016/j.cmi.2019.06.022
20. Fabiani M, Ferrante G, Minardi V, et al. Comparison of rubella simmunisation rates in immigrant and Italian women of childbearing age: Results from the Italian behavioral surveillance system PASSI (2011-2015). *PLoS One*. 2017;12(10):e0178122. doi:10.1371/journal.pone.0178122
21. Fortunato F, Iannelli G, Cozza A, et al. Local deprivation status and seasonal influenza vaccination coverage in adults ≥ 65 years residing in the Foggia municipality, Italy, 2009-2016. *J Prev Med Hyg*. 2019;59(4 Suppl 2):E51-E64. doi:10.15167/2421-4248/jpmh2018.59.4s2.1167
22. Fougère Y, El Houss S, Suris JC, et al. High coverage of hepatitis B vaccination and low prevalence of chronic hepatitis B in migrant children dictate a new catch-up vaccination strategy. *Vaccine*. 2018;36(30):4501-4506. doi:10.1016/j.vaccine.2018.06.010
23. Fozouni L, Weber C, Lindner AK, Rutherford GW. sImmunisation coverage among refugee children in Berlin. *J Glob Health*. 2019;9(1):010432. doi:10.7189/jogh.09.010432
24. Freidl GS, Tostmann A, Curvers M, et al. Immunity against measles, mumps, rubella, varicella, diphtheria, tetanus, polio, hepatitis A and hepatitis B among adult asylum seekers in the Netherlands, 2016. Vaccine. 2018;36(12):1664-1672. doi:10.1016/j.vaccine.2018.01.079
25. Fritschi N, Schmidt AJ, Hammer J, Ritz N; Swiss pediatric surveillance unit. Pediatric Tuberculosis Disease during Years of High Refugee Arrivals: A 6-Year National Prospective Surveillance Study. *Respiration*. 2021;100(11):1050-1059. doi:10.1159/000517029
26. Führer A, Eichner F, Stang A. Morbidity of asylum seekers in a medium-sized German city. *Eur J Epidemiol*. 2016;31(7):703-706. doi:10.1007/s10654-016-0148-4
27. Ganczak M, Bielecki K, Drozd-Dąbrowska M, et al. Vaccination concerns, beliefs and practices among Ukrainian migrants in Poland: a qualitative study. *BMC Public Health*. 2021;21(1):93.. doi:10.1186/s12889-020-10105-9

28. Georgakopoulou T, Horefti E, Vernardaki A, et al. Ongoing measles outbreak in Greece related to the recent European-wide epidemic. *Epidemiol Infect*. 2018;146(13):1692-1698. doi:10.1017/S0950268818002170
29. Glatman-Freedman A, Amir K, Dichtiar R, et al. Factors associated with childhood influenza vaccination in Israel: a cross-sectional evaluation. *Isr J Health Policy Res*. 2019;8(1):82. doi:10.1186/s13584-019-0349-x
30. Godefroy R, Chaud P, Ninove L, et al. Measles outbreak in a French Roma community in the Provence-Alpes-Côte d'Azur region, France, May to July 2017. *Int J Infect Dis*. 2018;76:97-101. doi:10.1016/j.ijid.2018.08.023
31. Gorman DR, Bielecki K, Willocks LJ, Pollock KG. A qualitative study of vaccination behaviour amongst female Polish migrants in Edinburgh, Scotland. *Vaccine*. 2019;37(20):2741-2747. doi:10.1016/j.vaccine.2019.03.073
32. Gorman DR, Bielecki K, Larson HJ, Willocks LJ, Craig J, Pollock KG. Comparing vaccination hesitancy in Polish migrant parents who accept or refuse nasal flu vaccination for their children. *Vaccine*. 2020;38(13):2795-2799. doi:10.1016/j.vaccine.2020.02.028
33. Habersaat KB, Pistol A, Stanescu A, et al. Measles outbreak in Romania: understanding factors related to suboptimal vaccination uptake. *Eur J Public Health*. 2020;30(5):986-992. doi:10.1093/eurpub/ckaa079
34. Hagstam P, Böttiger B, Winqvist N. Measles and rubella seroimmunity in newly arrived adult immigrants in Sweden. *Infect Dis (Lond)*. 2019;51(2):122-130. doi:10.1080/23744235.2018.1524583
35. Haider EA, Willocks LJ, Anderson N. Identifying inequalities in childhood immunisation uptake and timeliness in southeast Scotland, 2008-2018: A retrospective cohort study. *Vaccine*. 2019;37(37):5614-5624. doi:10.1016/j.vaccine.2019.07.080
36. Hardelid P, Rait G, Gilbert R, Petersen I. Factors associated with influenza vaccine uptake during a universal vaccination programme of preschool children in England and Wales: a cohort study. *J Epidemiol Community Health*. 2016;70(11):1082-1087. doi:10.1136/jech-2015-207014
37. Hudečková H, Stašková J, Mikas J, et al. Measles Outbreak in a Roma Community in the Eastern Region of Slovakia, May to October 2018. *Zdr Varst*. 2020;59(4):219-226. doi:10.2478/sjph-2020-0028
38. Hungerford D, Ibarz-Pavon A, Cleary P, French N. Influenza-associated hospitalisation, vaccine uptake and socioeconomic deprivation in an English city region: an ecological study. *BMJ Open*. 2018;8(12):e023275. doi:10.1136/bmjopen-2018-023275
39. Hungerford D, Vivancos R, Read JM, Iturriza-Gómara M, French N, Cunliffe NA. Rotavirus vaccine impact and socioeconomic deprivation: an interrupted time-series analysis of gastrointestinal disease outcomes across primary and secondary care in the UK. *BMC Med*. 2018;16(1):10. doi:10.1186/s12916-017-0989-z
40. Hvass AMF, Wejse C. High coverage of polio simmunisation program in refugees resettling in Denmark. A cross-sectional study of polio serology in newly arrived refugees. *Expert Rev Vaccines*. 2019;18(12):1317-1322. doi:10.1080/14760584.2019.1698953
41. Hvass AMF, Norredam M, Sodemann M, Thomsen MK, Christian W. Are refugees arriving in Denmark an under-immunised group for measles? A cross-sectional serology study. *Vaccine*. 2020;38(13):2788-2794. doi:10.1016/j.vaccine.2020.02.025
42. Iacoella C, Ralli M, Maggiolini A, Arcangeli A, Ercoli L. Acceptance of COVID-19 vaccine among persons experiencing homelessness in the City of Rome, Italy. *Eur Rev Med Pharmacol Sci*. 2021;25(7):3132-3135. doi:10.26355/eurrev_202104_25568

43. Jablonka A, Behrens GM, Stange M, et al. Tetanus and diphtheria immunity in refugees in Europe in 2015. *Infection*. 2017;45(2):157-164. doi:10.1007/s15010-016-0934-7
44. Jablonka A, Happle C, Wetzke M, et al. Measles, Rubella and Varicella IgG Seroprevalence in a Large Refugee Cohort in Germany in 2015: A Cross-Sectional Study. *Infect Dis Ther*. 2017;6(4):487-496. doi:10.1007/s40121-017-0169-7
45. Jackson C, Bedford H, Cheater FM, et al. Needles, Jabs and Jags: a qualitative exploration of barriers and facilitators to child and adult immunisation uptake among Gypsies, Travellers and Roma. *BMC Public Health*. 2017;17(1):254.. doi:10.1186/s12889-017-4178-y
46. Jenness SM, Aavitsland P, White RA, Winje BA. Measles vaccine coverage among children born to Somali immigrants in Norway. *BMC Public Health*. 2021;21(1):668. doi:10.1186/s12889-021-10694-z
47. Jones G, Haeghebaert S, Merlin B, et al. Measles outbreak in a refugee settlement in Calais, France: January to February 2016. *Euro Surveill*. 2016;21(11):30167. doi:10.2807/1560-7917.ES.2016.21.11.30167
48. Karasahin EF, Karasahin O, Kalkan IA. Results of Viral Hepatitis and Human Immunodeficiency Virus Screening in Afghan Irregular Migrants: A Cross-sectional Study (2011-2019). *Viral Hepatit Dergisi-Viral Hepatitis Journal*. 2021; 27:98-102.
49. Klok-Nentjes S, Tramper-Stranders GA, van Dam-Bakker EDM, Beldman J. Undocumented children in the Amsterdam region: an analysis of health, school, and living circumstances. *Eur J Pediatr*. 2018;177(7):1057-1062. doi:10.1007/s00431-018-3148-4
50. Köse Ş, Ödemiş I, Çelik D, Gireniz Tatar B, Akbulut I, Çiftdoğan DY. Hepatitis A, B, C and HIV seroprevalence among Syrian refugee children admitted to outpatient clinics. *Infez Med*. 2017;25(4):339-343.
51. Letley L, Rew V, Ahmed R, et al. Tailoring immunisation programmes: Using behavioural insights to identify barriers and enablers to childhood immunisations in a Jewish community in London, UK. *Vaccine*. 2018;36(31):4687-4692. doi:10.1016/j.vaccine.2018.06.028
52. Loiacono MM, Mahmud SM, Chit A, et al. Patient and practice level factors associated with seasonal influenza vaccine uptake among at-risk adults in England, 2011 to 2016: An age-stratified retrospective cohort study. *Vaccine X*. 2020;4:100054. doi:10.1016/j.jvacx.2020.100054
53. Louka C, Chandler E, Ranchor AV, et al. Asylum seekers' perspectives on vaccination and screening policies after their arrival in Greece and The Netherlands. *PLoS One*. 2019;14(12):e0226948. doi:10.1371/journal.pone.0226948
54. Mazzitelli M, Greco G, Serapide F, et al. Outcome of HBV screening and vaccination in a migrant population in southern Italy. *Infez Med*. 2021;29(2):236-241.
55. Mellou K, Chrisostomou A, Sideroglou T, et al. Hepatitis A among refugees, asylum seekers and migrants living in hosting facilities, Greece, April to December 2016. *Euro Surveill*. 2017;22(4):30448. doi:10.2807/1560-7917.ES.2017.22.4.30448
56. Mellou K, Silvestros C, Saranti-Papasaranti E, et al. Increasing childhood vaccination coverage of the refugee and migrant population in Greece through the European programme PHILOS, April 2017 to April 2018. *Euro Surveill*. 2019;24(27):1800326. doi:10.2807/1560-7917.ES.2019.24.27.1800326
57. Mueller-Hermelink M, Kobbe R, Methling B, et al. Universal screening for latent and active tuberculosis (TB) in asylum seeking children, Bochum and Hamburg, Germany, September 2015 to November 2016. *Euro Surveill*. 2018;23(12):17-00536. doi:10.2807/1560-7917.ES.2018.23.12.17-00536

58. Mylius M, Dreesman J, Zühlke C, Mertens E. Hemmnisse abbauen, Gesundheit fördern – Die Gesundheitsversorgung von Migrierten ohne Papiere im Rahmen eines Modellprojektes in Niedersachsen, 2016–2018 [Reduce obstacles, promote health-healthcare access for undocumented migrants within a pilot project in Lower Saxony, Germany (2016-2018)]. *Bundesgesundheitsblatt Gesundheitsforschung Gesundheitsschutz*. 2019;62(8):927-934. doi:10.1007/s00103-019-02972-4
59. Nakken CS, Skovdal M, Nellums LB, Friedland JS, Hargreaves S, Norredam M. Vaccination status and needs of asylum-seeking children in Denmark: a retrospective data analysis. *Public Health*. 2018;158:110-116. doi:10.1016/j.puhe.2018.02.018
60. Natan M, Kabha S, Yehia M, Hamza O. Factors That Influence Israeli Muslim Arab Parents' Intention to Vaccinate Their Children Against Influenza. *J Pediatr Nurs*. 2016;31(3):293-298. doi:10.1016/j.pedn.2015.12.014
61. Nidzvetska S, Rodriguez-Llanes JM, Aujoulat I, et al. Maternal and Child Health of Internally Displaced Persons in Ukraine: A Qualitative Study. *Int J Environ Res Public Health*. 2017;14(1):54. doi:10.3390/ijerph14010054
62. Norman FF, Comeche B, Martínez-Lacalzada M, et al. Seroprevalence of vaccine-preventable and non-vaccine-preventable infections in migrants in Spain. *J Travel Med*. 2021;28(4):taab025. doi:10.1093/jtm/taab025
63. Öztaş D, Kurt B, Akbaba M, Akyol M, Mollahaliloğlu S, Topaç O. Vaccination rates for Syrian population under temporary protection in Turkey. *Cent Eur J Public Health*. 2020;28(2):130-134. doi:10.21101/cejph.a5543
64. Perniciaro S, Imöhl M, van der Linden M. Invasive Pneumococcal Disease in Refugee Children, Germany. *Emerg Infect Dis*. 2018;24(10):1934-1936. doi:10.3201/eid2410.180253
65. Perry M, Townson M, Cottrell S, et al. Inequalities in vaccination coverage and differences in follow-up procedures for asylum-seeking children arriving in Wales, UK. *Eur J Pediatr*. 2020;179(1):171-175. doi:10.1007/s00431-019-03485-7
66. Perry M, McGowan A, Roberts R, Cottrell S. Timeliness and equity of infant pertussis vaccination in wales: Analysis of the three dose primary course. *Vaccine*. 2020;38(6):1402-1407. doi:10.1016/j.vaccine.2019.12.001
67. Pohl C, Mack I, Schmitz T, Ritz N. The spectrum of care for pediatric refugees and asylum seekers at a tertiary health care facility in Switzerland in 2015. *Eur J Pediatr*. 2017;176(12):1681-1687. doi:10.1007/s00431-017-3014-9
68. Rath B, Swenshon S, Haase K, et al. Using a mobile application to detect health needs among children and adolescents who are newly arrived migrants in Europe. *J Public Health (Oxf)*. 2019;41(4):840-849. doi:10.1093/pubmed/fdy191
69. Sane J, Sorvari T, Widerström M, et al. Respiratory diphtheria in an asylum seeker from Afghanistan arriving to Finland via Sweden, December 2015. *Euro Surveill*. 2016;21(5). doi: 10.2807/1560-7917.ES.2016.21.5.30126
70. Serre-Delcor N, Ascaso C, Soriano-Arandes A, et al. Health Status of Asylum Seekers, Spain. *Am J Trop Med Hyg*. 2018;98(1):300-307. doi:10.4269/ajtmh.17-0438
71. Shahbari NAE, Gesser-Edelsburg A, Davidovitch N, Brammli-Greenberg S, Grifat R, Mesch GS. Factors associated with seasonal influenza and HPV vaccination uptake among different ethnic groups in Arab and Jewish society in Israel. *Int J Equity Health*. 2021;20(1):201. doi:10.1186/s12939-021-01523-1

72. Staehelin C, Chernet A, Sydow V, et al. Seroprotection rates of vaccine-preventable diseases among newly arrived Eritrean asylum seekers in Switzerland: a cross-sectional study. *J Travel Med*. 2019;26(6):taz035. doi:10.1093/jtm/taz035
73. Stein Zamir C, Israeli A. Knowledge, Attitudes and Perceptions About Routine Childhood Vaccinations Among Jewish Ultra-Orthodox Mothers Residing in Communities with Low Vaccination Coverage in the Jerusalem District. *Matern Child Health J*. 2017;21(5):1010-1017. doi:10.1007/s10995-017-2272-5
74. Stein-Zamir C, Israeli A. Timeliness and completeness of routine childhood vaccinations in young children residing in a district with recurrent vaccine-preventable disease outbreaks, Jerusalem, Israel. *Euro Surveill*. 2019;24(6):1800004. doi:10.2807/1560-7917.ES.2019.24.6.1800004
75. Suppli CH, Dreier JW, Rasmussen M, et al. Sociodemographic predictors are associated with compliance to a vaccination-reminder in 9692 girls age 14, Denmark 2014-2015. *Prev Med Rep*. 2018;10:93-99. doi:10.1016/j.pmedr.2018.02.005
76. Tayfur I, Günaydin M, Suner S. Healthcare Service Access and Utilization among Syrian Refugees in Turkey. *Ann Glob Health*. 2019;85(1):42. doi:10.5334/aogh.2353
77. Taylor JEB, Surey J, MacLellan J, Francis M, Abubakar I, Stagg HR. Hepatitis B vaccination uptake in hard-to-reach populations in London: a cross-sectional study. *BMC Infect Dis*. 2019;19(1):372. doi:10.1186/s12879-019-3926-2
78. Tchidjou HK, Vescio MF, Serafinelli J, et al. Susceptibility to allergy in adoptive children: a cross-sectional study at "Bambino Gesù Children's Hospital". *Ital J Pediatr*. 2018;44(1):3. doi:10.1186/s13052-017-0440-2
79. Tessier E, Warburton F, Tsang C, et al. Population-level factors predicting variation in influenza vaccine uptake among adults and young children in England, 2015/16 and 2016/17. *Vaccine*. 2018;36(23):3231-3238. doi:10.1016/j.vaccine.2018.04.074
80. van den Heuvel R, Stammnitz A. Masern-Impfung bei Asylsuchenden in der Erstaufnahmeeinrichtung (EAE) Bielefeld: Impfquote 73%, Impfablehnung 21 [Immunization Against Measles at the Asylum Center Bielefeld, Germany: Vaccination Rate 73%, Refusal Rate 21]. *Gesundheitswesen*. 2020;82(5):e67-e71. doi:10.1055/a-1138-0603
81. Veronesi L, Colucci ME, Capobianco E, et al. Immunity status against poliomyelitis in young migrants: a seroprevalence study. *Acta Biomed*. 2019;90(9-S):28-34. doi:10.23750/abm.v90i9-S.8700
82. Vita S, Sinopoli MT, Fontanelli Sulekova L, et al. Vaccination campaign strategies in recently arrived migrants: experience of an Italian reception centre. *J Infect Dev Ctries*. 2019;13(12):1159-1164. doi:10.3855/jidc.11815
83. Vu F, Cavassini M, D'Acremont V, et al. Epidemiology of sexually transmitted infections among female sex workers in Switzerland: a local, exploratory, cross-sectional study. *Swiss Med Wkly*. 2020;150:w20357. doi:10.4414/smw.2020.20357
84. Ward C, Byrne L, White JM, Amirthalingam G, Tiley K, Edelstein M. Sociodemographic predictors of variation in coverage of the national shingles vaccination programme in England, 2014/15. *Vaccine*. 2017;35(18):2372-2378. doi:10.1016/j.vaccine.2017.03.042
85. Watkinson RE, Williams R, Gillibrand S, Sanders C, Sutton M. Ethnic inequalities in COVID-19 vaccine uptake and comparison to seasonal influenza vaccine uptake in Greater Manchester, UK: A cohort study. *PLoS Med*. 2022;19(3):e1003932. doi:10.1371/journal.pmed.1003932

86. Werber D, Hoffmann A, Santibanez S, Mankertz A, Sagebiel D. Large measles outbreak introduced by asylum seekers and spread among the insufficiently vaccinated resident population, Berlin, October 2014 to August 2015. *Euro Surveill*. 2017;22(34):30599. doi:10.2807/1560-7917.ES.2017.22.34.30599
87. Yakut N, Soysal S, Soysal A, Bakir M. Knowledge and acceptance of influenza and pertussis vaccinations among pregnant women of low socioeconomic status in Turkey. *Hum Vaccin Immunother*. 2020;16(5):1101-1108. doi:10.1080/21645515.2019.1689082
