# Supplementary material for: Access to Vaccination among Disadvantaged, Isolated and Difficult-to-Reach Communities in the WHO European Region: A Systematic Review
Source: Vaccines (Basel). 2022 Jun 28;10(7):1038. doi: 10.3390/vaccines10071038 (PMC9324407; doi:10.3390/vaccines10071038)
Supplement: Supplementary file 1 [file vaccines-10-01038-s001.zip › Supplementary S3 - Included COVID studies.pdf]

Supplementary File S3

Table B: Studies included in review showing vaccination uptake among different populations and countries (COVID-19 vaccine only) (n=19)

Author and year of study	Country	Study design and period of data collection	Study population, comparison group and sample size	Vaccine coverage/Uptake	Quality range
Ali-Saleh et al. (2022)[1]	Israel	Cross-sectional (Analytical)<br>16- 26 January 2021	Religious groups (Israeli Arabs)<br>- Adults (n=558)	**Vaccinated/Intends to be vaccinated (n=558):** 62.4% (348/558)<br><br>- **Religion**: Muslim (61.7%, 312/506), Christian (65.9%, 27/41), Druze (81.8%, 9/11)<br>- **Level of religiosity:** Secular (54.1%, 33/61), Partly religious (63.7%, 123/193), Religious (62.5%, 172/275), Orthodox (69.0%, 20/29)	Low
Bentivegna et al. (2022)[2]	Italy	Cross-sectional 2021	Migrants (Informal settlement dwellers - refugees, locals, homeless)<br>- Adults (n=160)	**Vaccination coverage by legal status**: Asylum seekers (28.9%), Irregulars (15.7%), Holders of other types of residency permits (38.5%)<br><br>**- Coverage by location:** Collatina (36%), Tiburtina (13%), Temini (31%)<br>**- Coverage of (irregular vs regular) by location:** Collatina (36% vs 36%), Tiburtina (11% vs 19%), Temini (23% vs 35%)<br>**- Coverage of (transiting vs resident) by location:** Collatina (11% vs 43%), Tiburtina (2% vs 29%)	Low
Blakeway et al. (2022)[3]	UK	Cohort (Retrospective)<br>1 March 2020 - 4 July 2021	Migrants (Ethnic minority)<br>- Adults (n=1,328 pregnant women)	28.5% (140/491) received at least 1 dose during pregnancy<br><br>**By Ethnicity**<br>**- At least 1st dose vaccinated (**n=140): Asian (12.1%), Afro-Caribbean (3.6%), Mixed (9.3%), Not reported (17.9%)<br>**- Did not receive a vaccine (n=1,188):** Asian (17.3%), Afro-Caribbean (8.5), Mixed (13.1%), Not reported (14.7%)<br><br>**By level of deprivation**<br>**- At least 1st dose vaccinated (n=140):** Most deprived (12.1%), Least deprived (3.6%),<br>- **Did not receive a vaccine (n=1,188)**: Most deprived (8.5%), Least deprived (8.6%)	Medium
Cook et al. (2022)[4]	UK	Cross-sectional (Prevalence)<br>January 2021 - March 2021	Migrants (Ethnic minority)<br>- Adults ≥16 years (n=1,058)	30.4% (322/1,058)Had at least one dose of COVID-19 vaccine<br>57.8% (612/1,058) Not yet had a vaccine	Low
Gaughan et al. (2022)[5]	UK	Cross-sectional (Analytical)<br>≥40 year old alive on 15 June 2021	Migrants (Ethnic minority)<br>Disadvataged ares (Deprivation)<br>- Adults ≥40 years, general population (n=24,094,186)	**Study population (n=24,094,186):** Bangladeshi (0.5%), Black African (1.3%), Black Caribbean (1.1%), Chinese (0.5%), Indian (2.6%), Pakistani (1.5%), White British (84.3%), White other (5%), Mixed ethnicity (1%), Other ethnicity (2.2%)<br><br>- **Received at least one vaccine dose**: 92% (22,143,218) by 15 June 2021 (UK vaccination programme began 8 December 2020)<br>**- Ethnic age-standardized vaccination rates by deprivation:** Most deprived - Black African (73.4%), Black Caribbean (62%), Bangladeshi (87.9%), Chinese (84.8%), Indian (90%), Pakistan (79.9%), white British (90.8%), white other ethnicities (74.5%)<br><br>- South Asian, black African and other ethnicities born in the UK had lower vaccination rates than their counterparts born abroad.<br>- All minority ethnic groups had lower probability of receiving a vaccine than white British, particularly black African (OR: 5.36; 95% CI:5.32-5.40) and black Caribbean (6.93; 6.87-6.98)	Low

Glampson et al. (2021)[6]	UK	Cohort (Retrospective) 2020 - 2021	Migrants (Ethnic minority)<br>- Adults ≥16 years, general population (n=2,183,939)	2,183,939 individuals eligible to receive COVID-19 vaccine,<br>**Vaccine offered (n=413,919)**: Accepted (94.12%), Declined and did not receive a vaccination (5.88%, 24,332/413,919)<br><br>**Rate of vaccine decline:**<br>- By ethnicity: Asian/Asian British (3.21%, 3867/120,291),Black/Black British (16.14%, 4337/26,870), Mixed ethnicity (10.39%, 895/8613), Other ethnicity (9.95%,2429/24,409), Unrecorded ethnicity (8.52%, 3016/35,419), White group (4.92%, 9788/187,090).<br>--- - Decline across all Black, Asian, and minority ethnic groups [6.39% (11,528/180,210)] compared with White group [4.92% (9788/187,090)]<br><br>**- By deprivation postcode:** Most deprived (13.5%, 1980/14,571), Least deprived (0.98%, 869/9609)<br>--- Strong negative association between deprivation and rate of declining vaccination (r=–0.94; P=.002)<br><br>**--- Highest rates of vaccine decline in most deprived areas:** 70-74 years (17.52%, 344/1963), 75-80 years (18.99%, 275/1448), ≥80years (25.91%, 524/2022), Clinically extremely vulnerable (19.17%, 377/1967), and Black/Black British (25.79%, 337/1967) communities	Meidum
Gorelik et al. (2022)[7]	Israel	Prevalence 2021	Religious groups (Arab, Ultra-orthodox and other Jews)<br>- All ages from 268 cities (n=8,507,723)	**Sample distribution (%), across 268 cities:** Arab (including Druze) (16.6%, 97 municipalities), Ultra-Orthodox (7.1%, 11 municipalities), General Jewish (63.2%, 154 municipalities), Mixed religion (13.1%, 6 municipalities)<br>**Vaccine coverage by ethnic/religion:** Arab (64.4%), Ultra-Orthodox Jewish (46.7%), General Jewish (80.1%), Mixed (62.7%)<br><br>- Vaccine coverage decreased with age and the difference between age groups was significant (p<0.001)	Low
Hall et al. (2021)[8]	UK	Cohort (Prospective, Multicentre) 7 Dec 2020 - 5 Feb 2021	Migrants (Ethnic minority)<br>- Adults (n=23,324, Health Care Workers)	23,324 participants<br>(Not vaccinated vs Vaccinated); OR (95%CI), ALL p-values were reported <0·0001<br><br>**By Ethnicity**<br>**- Asian (n=1,587):** (15.8% vs 84.2%); 0.62 (0.54–0.71)<br>**- Black (n=464):** (34.9% vs 65.1%); 0.22 (0.18–0.26)<br>**- Chinese (n=134):** (12.7% vs 87.3%); 0.80 (0·48–1.33)<br>**- Mixed race (n=356):** (19·4% vs 80·6%); 0·48 (0·37–0·63)<br>**- Other ethnic group (n=314):** (17·8% vs 82·2%); 0·53 (0·40–0·71)<br>**- Prefer not to say (n=45):** (22.2% vs 77·8%); 0·41 (0·20–0·82)<br>**- White (n=20,423):** (10.4% vs 89.6%); reference group<br><br>**By Deprivation:**<br>**- Most deprived** (n=2,634): (16·6% vs 83·4%); 0.50 (0.44–0.57)<br>**- Least deprived (n=5,614):** (9.0% vs 91.0%); reference group	Medium
Iacoella et al. (2021)[9]	Italy	Prevalence 2021 (Based on population census with age distribution was from 2018)	Migrant (Homeless)<br>- Adults (n=112)<br>- Africa (n=28), Asia (n=6), South America (n=5), Europe (n=73, including Italy, n=34)	**Willing to accept COVID-19 vaccine (n=112):** Yes (64.3%), Unsure (3.6%), Preferred not to be vaccinated (32.1%)<br>**- Willingness by gender (favourable):** Females (59.3%, Males (74.1%)<br>**- Willingness by age (yes):** 30-39 years (45.5%), 40-49 years (52.6%), 50-59 years (61.9%), 60-69 years (84.6%)<br>- Willingness to get vaccinated against COVID (~65%) similar with general population	Low

Kraft et al. (2022)[10]	Norway	Cross-sectional (Analytical)<br>28 December 2020 – 20 October 2021	Migrants (first and second generation, 41 country background)<br>- Adults ≥18 years , general population (n=4,264,370)<br><br>- Foreign-born (n= 689,540)<br>- Norwegian-born with foreign-born parents (n=57,153)<br>- Norwegian-born with Norwegian-born-parents (n= 3,518,308)	**Received at least one dose of a COVID-19 vaccine (%, n=4,264,370):** Foreign-born (73%, n=689,540), Norwegian-born individuals with foreign-born parents (82 %, n=57,153) Norwegian-born with Norwegian-born parents (93%, n=3 518 308)<br><br>**Large differences by country backgrounds:**<br>**- High coverage (>85 %):** Vietnam (93 %), Sri Lanka (91 %), Thailand (91 %), Denmark (89 %), the Philippines (89 %), India (88 %), the UK (88 %), Sweden (88 %) and Iran (87 %), Pakistan (86%), Iceland (86%)<br>**- Low coverage (<50%):** Latvia (44 %), Bulgaria (45 %), Romania (45 %), Poland (46 %) and Lithuania (47 %)<br><br>- **Influencing factors:** short time resident in Norway influences VCR<br>**- Lowest vaccination rates:** Bulgaria, Lithuania, Romania and Latvia had highest unvaccinated ORs	High
Martin et al. (2021)[11]	UK	Cross-sectional (Analytical)<br>All staff identified in Electronic Staff Record(ESR) on 3 February2021	Migrants (Ethnic minority)<br>- Adults (n=19,044, Health Care Workers)	Vaccinated (64.5%, n= 12,278) Unvaccinated (35.5%, n= 6,766)<br><br>Association with SARS-CoV-2 vaccine uptake.<br>(Vaccinated %, n/N); OR (95%CI), ALL p-values were reported <0·0001<br>**Vaccinated by ethnicity**<br>**--- White:** 70.9% (8,147/11,485); Reference group<br>**--- South Asian:** 58.5% (2,843/4,863); 0.58(0.54–0.62)<br>**--- Black:** 36.8% (499/1,357); 0.24(0.21–0.27)<br>**--- Other:** 609/1,038 (58.7)0.58(0.51–0.66)<br>**--- Not stated:** 180/301(59.8)0.61(0.48–0.77)<br><br>**Vaccinated by deprivation**<br>--- **Most deprived:** 1,688/2,940 (57.4)0.55(0.50–0.60)<br>**--- Least deprived:** 71.2% (3,274/4,597); Reference group	High
Muhsen et al. (2021)[12]	Israel	Cross-sectional (Analytical)<br>15 March 2020 - 27 February 2021	Religious groups (Jews, Arabs, Ultra-Orthodox Jewish)<br>- All ages, general population (n=482 million)	**- Immunised:** 1st dose (482 million), 2nd dose (351 million), representing a coverage of 53.2% and 38.8%, respectively<br>**-Vaccine uptake** lower in towns with mainly Arab and ultra-Orthodox Jewish populations compared to the general Jewish population, and it increased with improved residential SES	High
Nafilyan et al. (2021)[13]	UK	Cohort<br>≥70 years old alive on 15 March 2021	Migrants (Ethnic minority)<br>Disadvantaged areas (Deprivation)<br>- Adults ≥70 years, general population (n=6,655,672)	**Total receiving a vaccine:** Black (11.6%, 1,022/8,787), South Asian (15.4%, 2,339/15,199), Middle east/East Asia (13.3%, 922/6,946), White (15.4%, 171,453/1,110,544), Mixed/other (11.1%1,506/13,512),<br><br>**Lower income:** Black (11.7%, 455/3,904), South Asian (13.9%, 745/5,377), Middle East/East Asia (12%, 289/2,260), White (14.7%, 41,761/283,212), Mixed/other (10.1%, 459/4524)<br>**Higher income:** Black (12.9%, 111/863), South Asian (15,3%, 345/2,250), Middle East/East Asia (12.8%, 136/1,063), White (15.6%, 29993/192,710), Mixed/other (11.1%, 224/2,018)	High

Nguyen et al. (2022)[14]	UK	Cohort<br>24 March 2020 - 16 February 2021	Migrants (Ethnic minority)<br>- All ages (n = 1,254,294)	**Receiving a vaccine by ethnicity:** Black (11.6%, 1022/8787), South Asian: (15.4%, 2339/15,199), Middle east/East Asia: (13.3%, 922/6946), White: (15.4%, 171,453/1,110,544) Mixed/other: (11.1%, 1506/13,512)<br><br>**Received vaccine by deprivation:**<br>- **Lower income (Quartile 1):** Black: (11.7%. 455/3,904), South Asian: (13.9%, 745/5,377), Middle East/East Asia: (12.8%, 289/2,260), White: (14.7%, 41,761/283,212), Mixed/other: (10.1%, 459/4,524)<br>- **Higher income (Quartile 4):** Black: (12.9%, 111/863), South Asian: (15.3%, 345/2,250), Middle East/East Asia: (12.8, 136/1,063), White: (15.6%, 29993/192,710), Mixed/other: (11.1%, 224/2,018)	Low
Perry et al. (2021)[15]	Wales	Cross-sectional (Analytical)<br>≥50 years old alive on 31 March 2021 (UK), 5 April 2021 (Wales)	Migrants (Ethnic minority)<br>- Adults ≥50 years (n=1,256,412)	**Overall uptake of 1st dose of COVID-19 vaccine (8th December 2020 until mid-April 2021):** 92.1%<br><br>**Ethnic Sample distribution and vaccine uptake (%):** Asian (n= 14,001, 85.0%), Black (n= 3,954, 73.9%), White (n=1,134,610, 94.1%), Mixed (n=7,657, 82.5%), Other (n=2,815, 75.6%), Unknown (n=93,348, 72.0%)<br>- **Lowest uptake:** Black ethnicities (73.9%), Unknown ethnic groups (72%)<br>- **Largest inequality**: between ethnic groups, odds of being vaccinated 0.22(95 %CI 0.21–0.24) in Black compared to White ethnic groups.<br><br>**Deprivation sample distribution and vaccine uptake (%):** Most deprived (209,410, 89.7%), Least deprived (276,237, 94%)	Medium
Saban et al. (2021)[16]	Israel	Cross-sectional (Analytical)<br>20 December 2020 - 31 August 2021	Migrants (Ethnic minority) Disadvantaged areas (Deprivation)<br>- All ages, general population (n=8.214 million)	**Coverage by ethnic group:**<br>**- Jewish:** Dose 1 (59%), Dose 2 (54%), Dose 3 (17%)<br>**- Arab:** Dose 1 (55%), Dose 2 (50%), Dose 3 (14%)<br>**- Mixed:** Dose 1 (64%), Dose 2 (59%), Dose 3 (19%)<br><br>**Coverage by SES category**<br>**- Lowest SES:** Dose 1 (27%), Dose 2 (22%), Dose 3 (34%)<br>**- Highest SES:** Dose 1 (71%), Dose 2 (68%), Dose 3 (32%)<br><br>**Correlation between COVID-19 vaccine uptake and SES category:** Dose 1 (R2 = 0.4331), Dose 2 (R2 = 0.542), Dose 3 (R2 = 0.8416)	Low
Taubman-Ben-Ari et al. (2022)[17]	Israel	Cross-sectional (Analytical)<br>3 March - 7 April 2021	Religious groups (Jews, Arabs)<br>- Adults Pregnant women 19-46 years, general population (n=8,600)	Arab (n= 673), Jewish (n=167)<br><br>**- Recovery from Covid:** Jewish (6.5%), Arab (23.6%)<br>**- Covid-vaccination (not yet infected):** Jewish (93.6%), Arab (65. 6%),<br>**- Intention to receive vaccine (not yet vaccinated):** Jewish (85.7%), Arab (70.5%) Y3	Medium
Tessier et al. (2022) [18]	UK	Cross-sectional (Analytical)<br>8 December – 17 May 2021	Migrants (Ethnic minority)<br>- Adults ≥65 years, general population (n=61,967,781	**1st dose vaccination:** 49.4% (30,624,257 / 61,967,781)<br>**2nd dose vaccination:** 28.1% (17,360,045/61,967,781)<br><br>- The proportion of individuals unvaccinated varied by age, dose and region<br>- Highest predictive margin for not being vaccinated included among Black/African/Caribbean ethnic groups with a prevalence of 23. 9% unvaccinated	High
Watksinson et al. (2022) [19]	UK	Cohort (Retrospective)<br>1 December 2020 - 18 April 2021	Migrants (Ethnic minority)<br>- Adults ≥18 years (n=1,099,503)	**Covid-19 vaccine uptake:** 83.64% (919,636/1,099,503) of eligible individuals<br>**Ethnic minority:**<br>- Uptake lower compared to the White British group for 15 of 16 minority ethnic groups:<br>- Wide inequalities amongst the groups 'other Black background' (hazard ratio [HR] 0.42, 95% CI 0.40 to 0.44), Black African (HR 0.43, 95% CI 0.42 to 0.44), Arab (HR 0.43, 95% CI 0.40 to 0.48), and Black Caribbean (HR 0.43, 95% CI 0.42 to 0.45)	Medium

References List – Included COVID studies

1. Ali-Saleh O, Bord S, Basis F. Low Response to the COVID-19 Vaccine Among the Arab Population in Israel: Is It a Cultural Background, or a Systemic Failure, or Maybe Both? *J Racial Ethn Health Disparities*. 2022;1-10. doi:10.1007/s40615-021-01220-3
2. Bentivegna E, Di Meo S, Carriero A, Capriotti N, Barbieri A, Martelletti P. Access to COVID-19 Vaccination during the Pandemic in the Informal Settlements of Rome. *Int J Environ Res Public Health*. 2022;19(2):719. doi:10.3390/ijerph19020719
3. Blakeway H, Prasad S, Kalafat E, et al. COVID-19 vaccination during pregnancy: coverage and safety. *Am J Obstet Gynecol*. 2022;226(2):236.e1-236.e14. doi:10.1016/j.ajog.2021.08.007
4. Cook EJ, Elliott E, Gaitan A, et al. Vaccination against COVID-19: Factors That Influence Vaccine Hesitancy among an Ethnically Diverse Community in the UK. *Vaccines (Basel)*. 2022;10(1):106. doi:10.3390/vaccines10010106
5. Gaughan CH, Razieh C, Khunti K, et al. COVID-19 vaccination uptake amongst ethnic minority communities in England: a linked study exploring the drivers of differential vaccination rates. *J Public Health (Oxf)*. 2022;fdab400. doi:10.1093/pubmed/fdab400
6. Glampson B, Brittain J, Kaura A, et al. Assessing COVID-19 Vaccine Uptake and Effectiveness Through the North West London Vaccination Program: Retrospective Cohort Study. *JMIR Public Health Surveill*. 2021;7(9):e30010. doi:10.2196/30010
7. Gorelik Y, Anis E, Edelstein M. Inequalities in initiation of COVID19 vaccination by age and population group in Israel- December 2020-July 2021. *Lancet Reg Health Eur*. 2022;12:100234. doi:10.1016/j.lanepe.2021.100234
8. Hall VJ, Foulkes S, Saei A, et al. COVID-19 vaccine coverage in health-care workers in England and effectiveness of BNT162b2 mRNA vaccine against infection (SIREN): a prospective, multicentre, cohort study. *Lancet*. 2021;397(10286):1725-1735. doi:10.1016/S0140-6736(21)00790-X
9. Iacoella C, Ralli M, Maggiolini A, Arcangeli A, Ercoli L. Acceptance of COVID-19 vaccine among persons experiencing homelessness in the City of Rome, Italy. Eur Rev Med Pharmacol Sci. 2021;25(7):3132-3135. doi:10.26355/eurrev_202104_25568
10. Kraft KB, Godøy AA, Vinjerui KH, Kour P, Kjøllesdal MKR, Indseth T. COVID-19 vaccination coverage by immigrant background. Vaksinasjonsdekning mot covid-19 etter innvandrerbakgrunn. *Tidsskr Nor Laegeforen*. 2021;141(2):10.4045/tidsskr.21.0799. doi:10.4045/tidsskr.21.0799
11. Martin CA, Marshall C, Patel P, et al. SARS-CoV-2 vaccine uptake in a multi-ethnic UK healthcare workforce: A cross-sectional study. *PLoS Med*. 2021;18(11):e1003823. doi:10.1371/journal.pmed.1003823
12. Muhsen K, Na'aminh W, Lapidot Y, et al. A nationwide analysis of population group differences in the COVID-19 epidemic in Israel, February 2020-February 2021. *Lancet Reg Health Eur*. 2021;7:100130. doi:10.1016/j.lanepe.2021.100130
13. Nafilyan V, Dolby T, Razieh C, et al. Sociodemographic inequality in COVID-19 vaccination coverage among elderly adults in England: a national linked data study. *BMJ Open*. 2021;11(7):e053402. doi:10.1136/bmjopen-2021-053402
14. Nguyen LH, Joshi AD, Drew DA, et al. Self-reported COVID-19 vaccine hesitancy and uptake among participants from different racial and ethnic groups in the United States and United Kingdom. *Nat Commun*. 2022;13(1):636. doi:10.1038/s41467-022-28200-3
15. Perry M, Akbari A, Cottrell S, et al. Inequalities in coverage of COVID-19 vaccination: A population register based cross-sectional study in Wales, UK. *Vaccine*. 2021;39(42):6256-6261. doi:10.1016/j.vaccine.2021.09.019
16. Saban M, Myers V, Ben-Shetrit S, Wilf-Miron R. Socioeconomic gradient in COVID-19 vaccination: evidence from Israel. *Int J Equity Health*. 2021;20(1):242. doi:10.1186/s12939-021-01566-4

17. Taubman-Ben-Ari O, Weiss E, Abu-Sharkia S, Khalaf E. A comparison of COVID-19 vaccination status among pregnant Israeli Jewish and Arab women and psychological distress among the Arab women. *Nurs Health Sci*. 2022;10.1111/nhs.12929. doi:10.1111/nhs.12929
18. Tessier E, Rai Y, Clarke E, et al. Characteristics associated with COVID-19 vaccine uptake among adults aged 50 years and above in England (8 December 2020-17 May 2021): a population-level observational study. *BMJ Open*. 2022;12(3):e055278. doi:10.1136/bmjopen-2021-055278
19. Watkinson RE, Williams R, Gillibrand S, Sanders C, Sutton M. Ethnic inequalities in COVID-19 vaccine uptake and comparison to seasonal influenza vaccine uptake in Greater Manchester, UK: A cohort study. PLoS Med. 2022;19(3):e1003932. doi:10.1371/journal.pmed.100393
